# Chk1 KA1 domain auto-phosphorylation stimulates biological activity and is linked to rapid proteasomal degradation

**DOI:** 10.1038/s41598-018-35616-9

**Published:** 2018-12-03

**Authors:** Eun-Yeung Gong, Beatriz Hernández, Jessica Hernández Nielsen, Veronique A. J. Smits, Raimundo Freire, David A. Gillespie

**Affiliations:** 1 Unidad de Investigación, Hospital Universitario de Canarias, Ofra s/n, La Cuesta, La Laguna, 38320 Tenerife, Spain; 2 Instituto de Tecnologías Biomédicas, Centro de Investigaciones Biomédicas de Canarias, Facultad de Medicina, Campus Ciencias de la Salud, Universidad de La Laguna, La Laguna, 38071 Tenerife, Spain; 3 Present Address: Nano-Bio Resources Center, Sookmyung Women’s University, Chungpa-Dong 2-Ka, Yongsan-ku, Seoul 04310 Republic of Korea

## Abstract

The DNA damage-activated protein kinase Chk1 is known to undergo auto-phosphorylation, however the sites and functional significance of this modification remain poorly understood. We have identified two novel Chk1 auto-phosphorylation sites, threonines 378 and 382 (T378/382), located in a highly conserved motif within the C-terminal Kinase Associated 1 (KA1) domain. T378/382 occur within optimal consensus Chk1 phosphorylation motifs and substitution with phospho-mimetic aspartic acid residues results in a constitutively active mutant Chk1 kinase (Chk1-DD) that arrests cell cycle progression in G2 phase of the cell cycle in the absence of DNA damage. Remarkably, the mutant Chk1-DD protein is also subject to very rapid proteasomal degradation, with a half-life approximately one tenth that of wild-type Chk1. Consistent with this, T378/T382 auto-phosphorylation also accelerates the proteasomal degradation of constitutively active Chk1 KA1 domain structural mutants. T378/382 auto-phosphorylation and accelerated degradation of wild-type Chk1 occurs at low levels during unperturbed growth, but surprisingly, is not augmented in response to genotoxic stress. Taken together, these observations demonstrate that Chk1 T378/T382 auto-phosphorylation within the KA1 domain is linked to kinase activation and rapid proteasomal degradation, and suggest a non-canonical mechanism of regulation.

## Introduction

The serine-threonine protein kinase Chk1 is a key regulator of the DNA damage and replication checkpoints in vertebrate cells^1^. Chk1 is activated in response to a wide variety of genotoxic insults and triggers multiple downstream responses according to the nature of the genomic damage induced^2^. In response to DNA double strand breaks (DSBs), Chk1 arrests cells in G2 phase to delay mitosis whilst simultaneously promoting DNA repair by homologous recombination^1^. During DNA synthesis inhibition, Chk1 blocks the onset of mitosis in cells with incompletely replicated DNA whilst also acting to stabilise stalled replication forks and inhibit late replication origin firing^1^. Collectively, these canonical interphase DNA structure checkpoint responses promote genomic stability and cell survival under conditions of genotoxic stress. Chk1 also plays less well-characterised roles in the spindle^3^ and abscission^4^ checkpoints that monitor the fidelity of mitosis and in regulating gene expression by modulating chromatin structure^5^.

Activation of Chk1 in response to genotoxic stress requires phosphorylation of multiple serine-glutamine (SQ) residues within the C-terminal regulatory region that is catalysed by the upstream regulatory kinase ATR^1^, most prominently serine 317 (S317) and serine 345 (S345). Phosphorylation of S345 in particular is critical for Chk1 activation, as substitution of this single site with a non-phosphorylatable alanine residue results in a complete loss of biological function in response to genotoxic stress^6^. Exactly how S345 phosphorylation leads to Chk1 activation remains unclear, however the C-terminal regulatory region of Chk1 can bind to the kinase domain and repress its activity^1^. Recently crystallographic analysis has demonstrated that this intramolecular interaction is mediated specifically by a KA1 domain structure contained within the regulatory region^7^. One possibility therefore is that S345 phosphorylation dissociates the intramolecular interaction between the kinase and KA1 domains, thus de-repressing catalytic activity and enabling Chk1 to phosphorylate its downstream substrates^1,7^. Although many observations are generally consistent with this model, the exact molecular details remain to be established.

Chk1 is also known to undergo auto-phosphorylation, however the sites and potential regulatory significance of this modification have not been as thoroughly explored. Serine 296 (S296) has been identified as a Chk1 auto-phosphorylation site^8,9^. Modification of this residue is stimulated by DNA damage, contingent on prior modification of S317/S345 by ATR^9^, and plays an important role in dispersing Chk1 through the nucleoplasm and targeting it to its substrate Cdc25A via interaction with 14-3-3 gamma^8^. In addition, replacement of S296 with a non-phosphorylatable alanine impairs checkpoint proficiency^10^. Taken together, these data suggest that auto-phosphorylation of S296 contributes to the conventional mechanism of Chk1 activation by genotoxic stress in collaboration with ATR. Curiously, the amino acid sequence surrounding S296 does not conform to the optimal consensus for Chk1 phosphorylation defined for *tran*s-phosphorylated substrates, however this may be explained by the fact that S296 auto-phosphorylation is catalysed only in *cis* by an intramolecular mechanism^9^.

Recovery from a DNA damage-induced checkpoint arrest requires de-activation of Chk1 and selective destruction of active, S345-phosphorylated Chk1 by polyubiquitination and proteasomal degradation plays an important role in this process^11^. Ubiquitination of Chk1 can be mediated by two distinct Cullin-RING ligase (CRL) complexes, CRL1–SKP1–Fbx6 and CRL4–DDB1–CDT2^12,13^. These distinct complexes are thought to promote Chk1 degradation in different cellular compartments: CRL1–SKP1–Fbx6 in the cytoplasm^12^, and CRL4–DDB1–CDT2 in the nucleoplasm^13^. Interestingly, in the case of CRL1–SKP1–Fbx6, Chk1 degradation was shown to be mediated via interaction of Fbx6 with a degron-like region in the C-terminal regulatory domain^12^. Although the putative degron was not mapped to fine resolution, it was assigned to a region (amino acids 368–421) now known to be located within the KA1 domain^7^. Whether CRL4–DDB1–CDT2 and potentially other ubiquitin ligase complexes can also interact with this putative degron remains unknown.

Activation of Chk1 by ATR-mediated S345 phosphorylation in response to DNA damage or DNA synthesis inhibition is thought to occur within a multi-component complex on chromatin where the primary DNA structure lesions are sensed^1^. However, it is less clear how Chk1 is regulated to function in other processes such as the spindle and abscission checkpoints^3,4^, since these can be active in the absence of exogenous genotoxic stress and occur at non-canonical subcellular locations such as the centromeres and mid-body respectively. Further, it has been proposed that Chk1 can be active in undamaged cells to promote gene expression via phosphorylation of threonine 11 (T11) in histone H3 without triggering its acute checkpoint functions^5^. It appears therefore that not all aspects of Chk1 regulation may be fully explicable in terms of ATR-mediated S345 phosphorylation.

Here we report the identification of two novel Chk1 auto-phosphorylation sites, threonines 378 and 382 (T378/382), that are located within a highly conserved amino acid motif within the C-terminal KA1 domain and which conform to the optimal consensus sequence for Chk1 phosphorylation. Using a Chk1 mutant bearing phospho-mimetic amino acid substitutions of T378/382, in combination with previously described structural mutants bearing point mutations specifically within the KA1 domain that confer enhanced kinase activity and constitutive biological activity on Chk1^14^, we show that auto-phosphorylation of these sites is linked both to Chk1 activation and degradation. Strikingly, wild-type Chk1 undergoes T378/382 auto-phosphorylation at low levels during unperturbed cell cycle progression however this is not stimulated by genotoxic stress and therefore does not appear to be linked to the canonical mechanism of Chk1 activation.

## Results

### Chk1 threonines 378 and 382 (T378/382) are novel auto-phosphorylation sites

**Figure 1:**
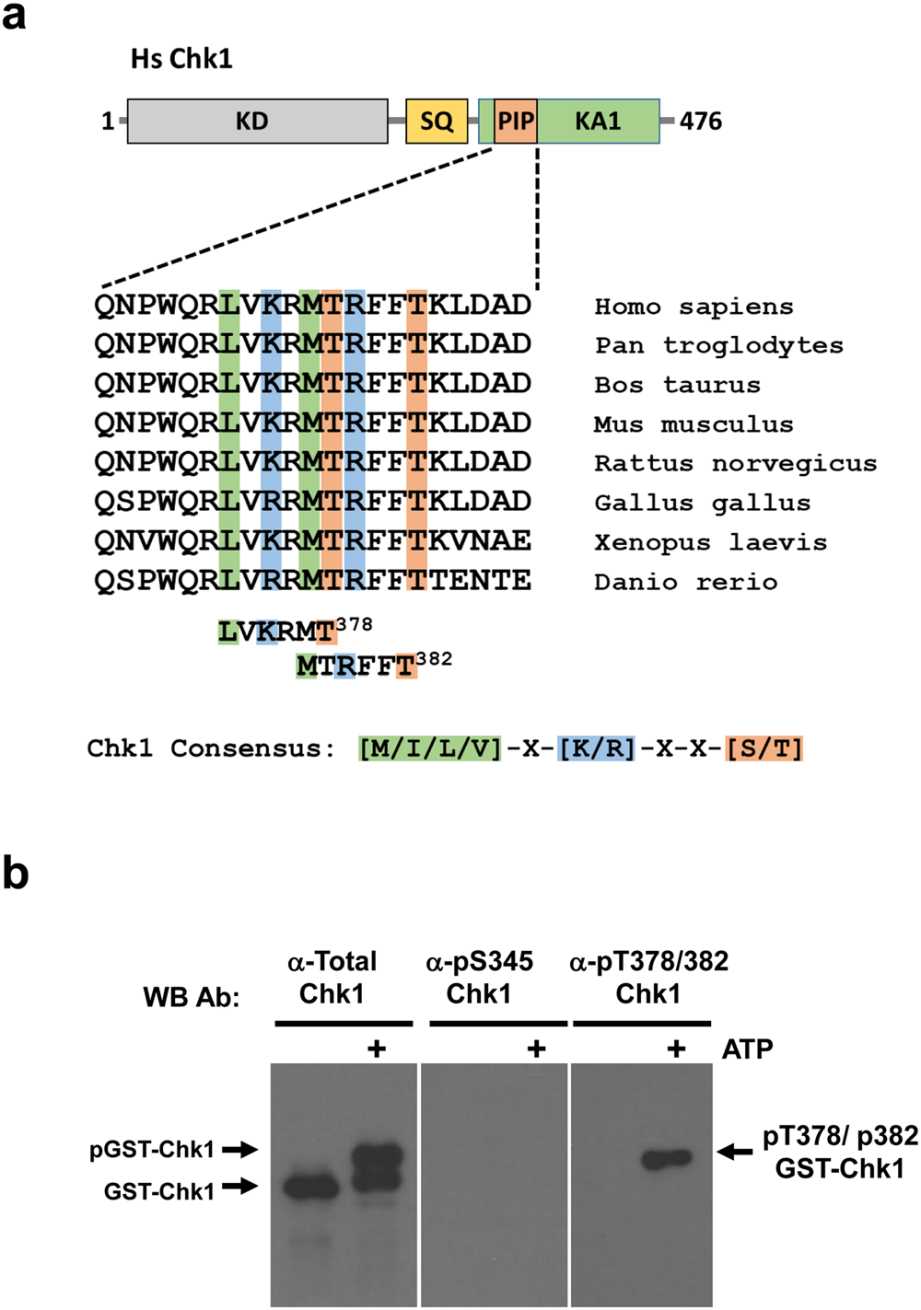
Identification of T378/382 as novel sites of Chk1 auto-phosphorylation. (**a**) Alignment of vertebrate Chk1 sequences from humans to zebrafish across a short region within the C-terminal kinase-associated 1 (KA1) domain which has previously been termed either the “PCNA-interacting (PIP) motif^16^” or “Conserved motif (CM)^12^”. ScanProsite analysis using the optimal consensus sequence for Chk1 phosphorylation [M/I/L/V]-X-[K/R]-X-X-[S/T] identified T378/382 as potential auto-phosphorylation sites. (**b**) 150 ng of recombinant Human GST-Chk1 was incubated in Chk1 kinase buffer plus or minus 100 μM ATP for 30 mins at 30 °C. After incubation 50 ng portions were resolved in triplicate by SDS-PAGE gel and the replicates analysed by western blotting using antibodies against total Chk1, or specific for Chk1 phosphorylated on S345 or T378/382.

To identify potential sites of Chk1 auto-phosphorylation, we scanned the amino acid sequence of human Chk1 for serine or threonine residues that occur within the Chk1 consensus specificity sequence^15^, [M/I/L/V]-X-[K/R]-X-X-[S/T], using ScanProsite (https://prosite.expasy.org/scanprosite), a bioinformatic tool for amino acid motif detection. This revealed two closely spaced threonine residues contained within partially overlapping optimal consensus Chk1 phosphorylation sites at positions 378 and 382 in the C-terminal regulatory domain, **L**-V-**K**-R-M-**T**^**378**^ and **M**-T-**R**-F-F-**T**^**382**^ (Fig. 1a, hereafter referred to as T378/382). Interestingly, these sites occur within a motif which has been highly conserved during evolution from human to zebrafish and which has previously been implicated both in Chk1 degradation and PCNA binding (Fig. 1a)^12,16^.

To determine if T378/382 could serve as sites of auto-phosphorylation, we generated a phospho-specific antiserum using a 12 amino acid synthetic peptide containing both phospho-T378 and phospho-T382 as immunogen. This antiserum was purified by affinity chromatography to obtain antibodies specific for the phospho-peptide (see Materials and Methods for further details). To test for Chk1 T378/382 auto-phosphorylation, we incubated recombinant full-length GST-Chk1 purified from insect cells in kinase assays with or without ATP (Fig. 1b). Replicate western blots were prepared and probed with antibodies specific either for total Chk1 or Chk1 phosphorylated at S345 or T378/382. As shown in Fig. 1b (left panel), a significant proportion of recombinant GST-Chk1 was capable of auto-phosphorylation as judged by an electrophoretic mobility shift detected by total Chk1 antibody that occurred only in the presence of ATP. Strikingly, this shifted form was strongly reactive with the anti-pT378/382 antiserum (Fig. 1b, right panel), indicating that these residues are indeed sites of auto-phosphorylation, at least *in vitro*. In marked contrast, the shifted form of Chk1 was not recognised by an antibody specific for phosphorylation of S345 (Fig. 1b, middle panel), a site that occurs in a distinct amino acid context and which is normally modified by ATR^6^.

### Phospho-mimetic aspartic acid substitutions at T378/382 confer constitutive biological activity on Chk1 associated with rapid proteasomal degradation

**Figure 2:**
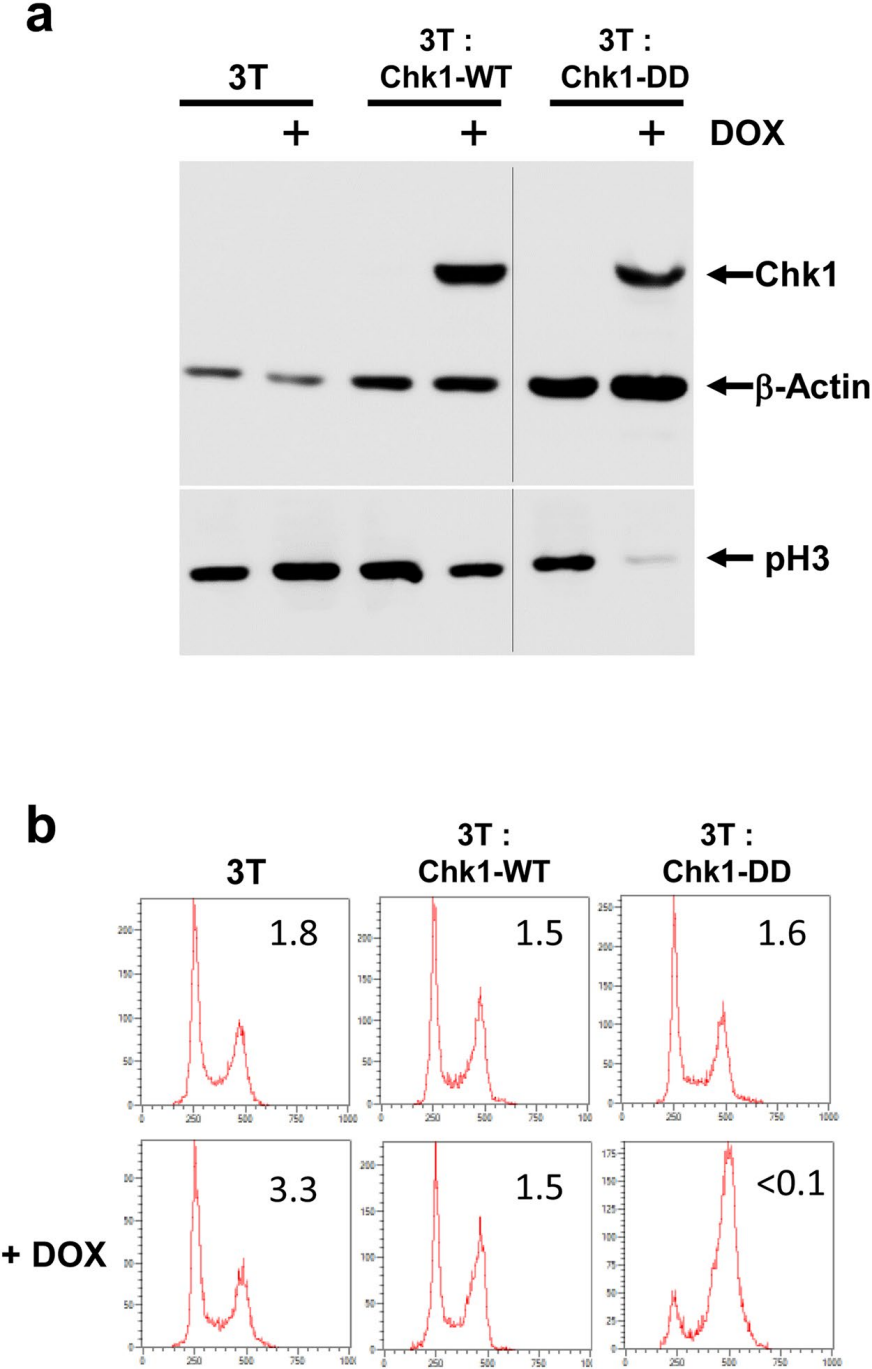
The phospho-mimetic auto-phosphorylation site mutant Chk1-DD induces G2 arrest in the absence of DNA damage. (**a**) 3T, 3T: Chk1-WT and 3T: Chk1-DD DT40 cell lines were treated with DOX for 16 hours and cell extracts were prepared from half of the cultures. 30 μg of each extract was then analysed by western blotting using indicated antibodies. Samples shown in the upper and lower panels were resolved on the same western blots respectively (see Supplementary Information for original images). (**b**) The remaining portion of each culture was fixed in ethanol, stained with antibody against phospho-serine 10 histone H3 (pH3, a marker of mitotic cells) and propidium iodide (PI), and analysed by flow cytometry for DNA content and the percentage of mitotic (pH3 positive) cells (inset values).

To investigate the potential functional significance of T378/382 auto-phosphorylation, we substituted both threonines with phospho-mimetic aspartic acid residues to generate the mutant Chk1-DD. Chk1-DD was introduced into the doxycycline (DOX)-regulated expression cassette in the Chk1 knockout DT40 cell line, 3T, as described previously^14^. Cultures of 3T, 3T: Chk1-WT, and 3T: Chk1-DD cells were treated for 16 hours with DOX or vehicle control, harvested, and analysed western blotting and flow cytometry. As shown in Fig. 2a, DOX treatment induced the expression of both Chk1-WT and Chk1-DD proteins, however the cell cycle distribution of the treated cultures was strikingly different. DOX had little or no effect on cell cycle progression in the 3T or 3T: Chk1-WT cultures, however cells expressing Chk1-DD were observed to accumulate in the G2/M phase of the cell cycle as judged by DNA content analysis (Fig. 2b). Furthermore, the DOX-treated 3T: Chk1-DD culture contained very few histone H3 phospho-serine 10-positive (pH3) mitotic cells (Fig. 2b), and showed only low pH3 levels in western blotting (Fig. 2a), indicating that the cells were arrested in the G2 phase of the cell cycle rather than mitosis. Thus, phospho-mimetic substitutions of the T378/382 auto-phosphorylation sites activate Chk1, as previously described for structural mutations within the KA1 domain^14^.

**Figure 3:**
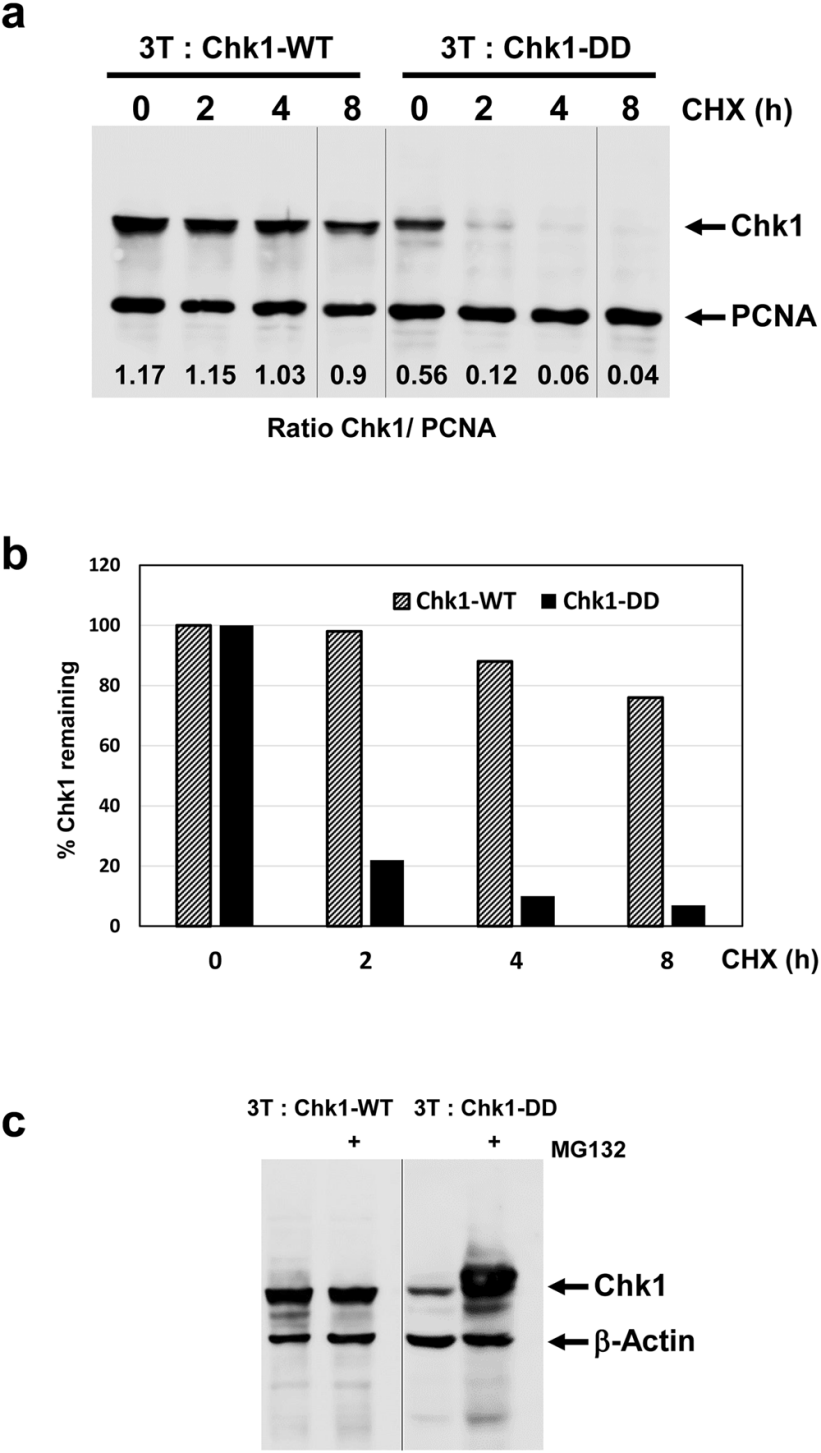
Chk1-DD is subject to rapid proteasomal degradation. (**a**) 3T: Chk1-WT and 3T: Chk1-DD DT40 cell lines were treated with DOX for 16 hours to induce Chk1 protein expression and then further treated with cycloheximide (CHX) to block protein synthesis for the indicated times. Cell extracts were prepared, 30 μg resolved by SDS-PAGE, and analysed by western blotting using the indicated antibodies. Samples were resolved on the same western blot (see Supplementary Information for original images). (**b**) Quantification of the data shown in (**a**), the percentage Chk1 protein remaining at each time point was calculated using PCNA to correct for variations in loading. Control values for t = 0 are converted to 100% to facilitate comparison of relative protein stability. (**c**) 3T: Chk1-WT and 3T: Chk1-DD cell lines were treated for 16 hours with DOX plus or minus MG132 to inhibit protein degradation via the proteasome. Cell extracts were prepared, 30 μg resolved by SDS-PAGE, and analysed by western blotting using the indicated antibodies. Samples were resolved on two separate western blots (see Supplementary Information for original images).

During our initial analyses we noticed that the expression of the Chk1-DD was reduced compared to Chk1-WT (Fig. 3a). Given that both proteins are expressed from the same TET-regulated expression cassette, we considered that the Chk1-DD mutant protein might be less stable than Chk1-WT. To test this, we incubated DOX-induced cells expressing Chk1-WT and Chk1-DD with cycloheximide to inhibit protein synthesis for 2, 4, and 8 hours, and monitored the rate at which the Chk1 proteins were degraded. As shown in Fig. 3a,b, Chk1-WT was relatively stable, with a half-life in excess of 8 hours, whereas strikingly, Chk1-DD was rapidly degraded, with a half-life of less than 2 hours. Importantly, when we treated cells with DOX and MG132, to inhibit the proteasome, we observed that the expression of Chk1-DD increased to a level comparable to or greater than Chk1-WT (Fig. 3c). Thus, the rapid degradation of Chk1-DD is mediated, at least in large part, via the proteasome.

### Chk1 T378/382 phosphorylation is linked to rapid proteasomal degradation *in vivo*

**Figure 4:**
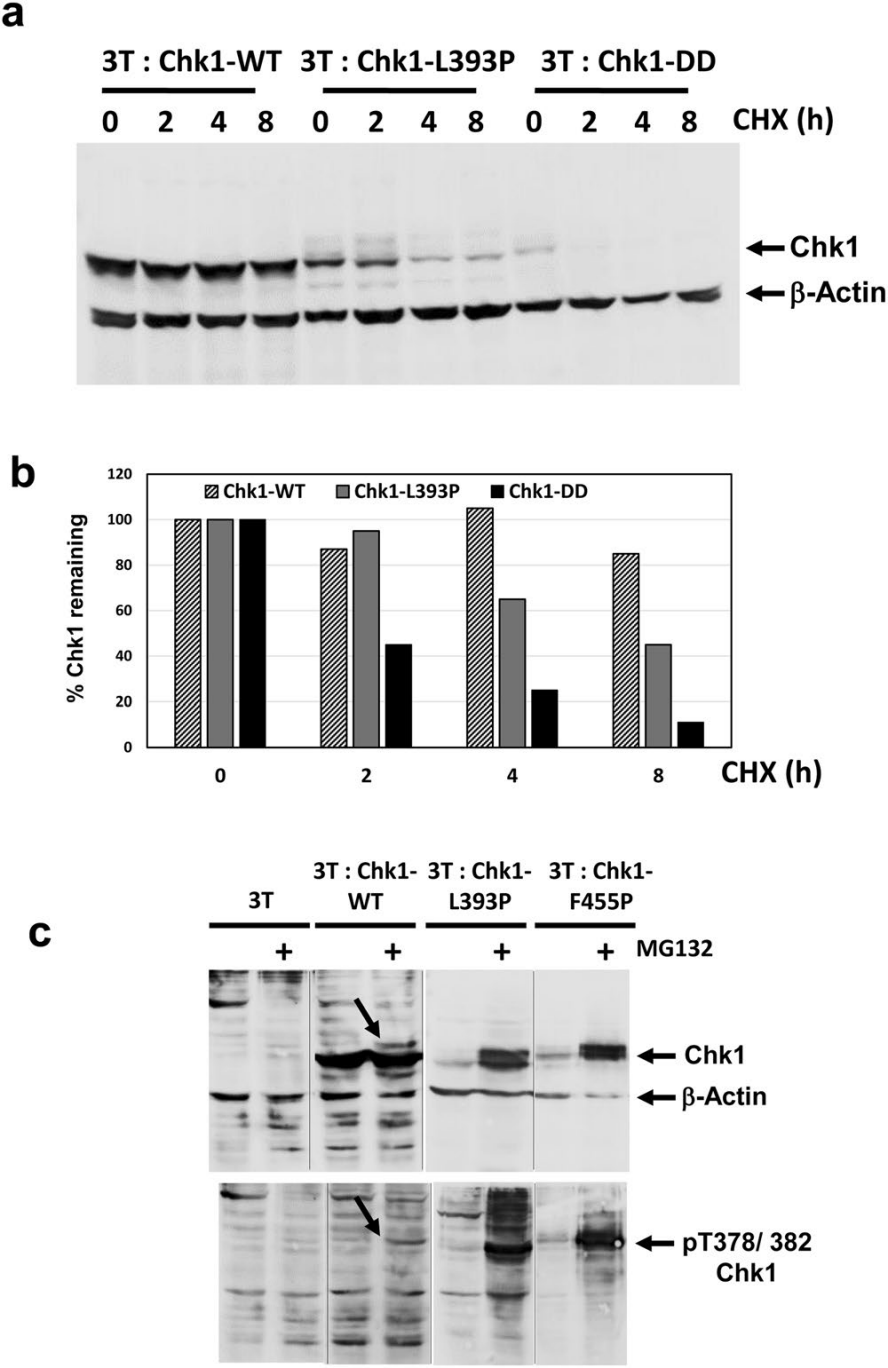
p378/382-auto-phosphorylated Chk1 is selectively degraded by the proteasome. (**a**) 3T: Chk1-WT, 3T: Chk1-L393P and 3T: Chk1-DD cell lines were treated with DOX for 16 hours to induce the expression of the corresponding proteins and then further treated for the indicated times with cycloheximide (CHX) to block protein synthesis. Cell extracts were prepared, 30 μg resolved by SDS-PAGE, and analysed by western blotting using the indicated antibodies. Samples were resolved on the same western blot (see Supplementary Information for original images). (**b**) Quantification of the data shown in (**a**), the percentage Chk1 protein remaining at each time point was calculated using B- Actin to correct for variations in loading. Control values for t = 0 are converted to 100% to facilitate comparison of relative protein stability. (**c**) The indicated DT40 cell lines were treated with DOX in the presence or absence of MG132 to induce the expression of Chk1-WT, and the active KA1 domain structural mutants Chk1-L393P and Chk1-F455P under conditions where the proteasome is active or inhibited by MG132, parental 3T cells serve as a negative control. Cell extracts were prepared, 30 μg resolved by SDS-PAGE, and analysed by western blotting using the indicated antibodies. Auto-phosphorylation of Chk1-WT, which is significantly weaker than the Chk1-L393P and Chk1-F455P mutants, is indicated by arrows in each panel. Samples in the upper and lower panels respectively are derived from two western blots run, processed and imaged in parallel in each case (see Supplementary Information for original images).

Previously, we reported that constitutively active KA1 domain mutants of Chk1 auto-phosphorylated at T378/382 vigorously when immunoprecipitated from cells and incubated in a kinase assay *in vitro*^14^. Perplexingly however, we were initially unable to detect T378/382 phosphorylation of these same mutant proteins in cell extracts using the anti-pT378/382 antibody (data not shown). In view of the instability of the phospho-mimetic Chk1-DD mutant, we considered that there might be a link between T378/382 auto-phosphorylation and proteasomal degradation *in vivo*. To test this, we first investigated the stability of a representative constitutively active KA1 mutant, Chk1-L393P^14^, in a cycloheximide chase experiment. Strikingly, this revealed that Chk1-L393P was also more rapidly degraded than Chk1-WT, although not as rapidly as Chk1-DD (Fig. 4a,b). Next, we examined the effect of proteasome inhibition with MG132 on the expression level and T378/382 phosphorylation of Chk1-L393P. As with Chk1-DD, we observed a substantial increase in the expression level of Chk1-L393P protein after MG132 treatment (Fig. 4c, upper panel), and strikingly, this was associated with the appearance of a very strong T378/382 phosphorylation signal (Fig. 4c, lower panel). Very similar results were obtained with a second KA1 domain mutant, Chk1-F455P, indicating that selective stabilisation of the T378/382-auto-phosphorylated form was not specific to Chk1-L393P (Fig. 4c). We deduce from these results that active KA1 domain mutants of Chk1 do undergo phosphorylation on T378/382 *in vivo*, but that the modified proteins are not readily detected because they are subject to very rapid proteasomal degradation. In comparison, MG132 treatment induced a much weaker T378/382 phosphorylation signal for Chk1-WT, consistent with its relatively slower rate of degradation (Fig. 4c).

### Active KA1 domain mutants of Chk1 auto-phosphorylate at T378/382 *in vivo*

**Figure 5:**
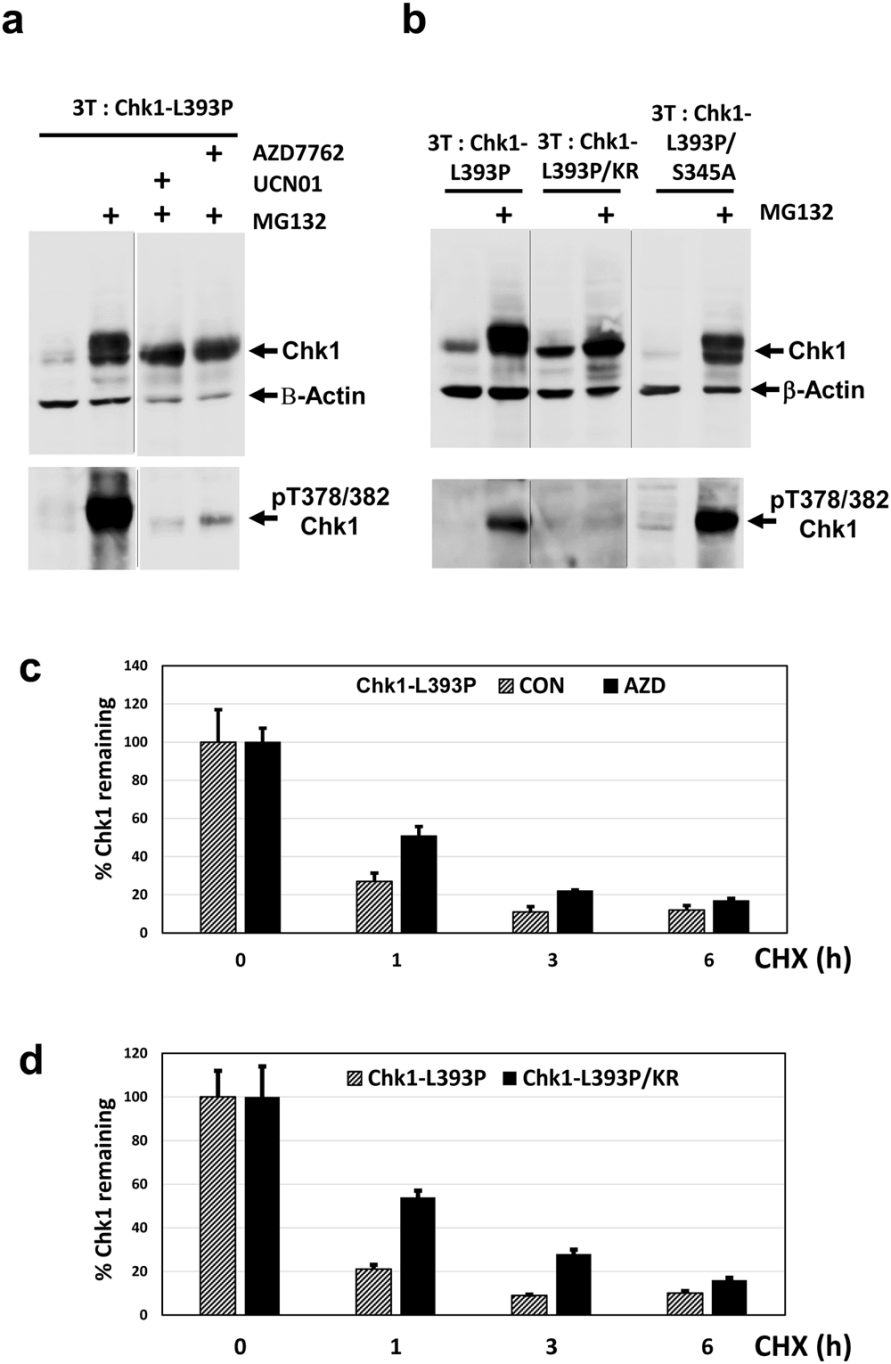
Chk1 378/382 auto-phosphorylation accelerates proteasomal degradation. (**a**) 3T: Chk1-L393P DT40 cells were treated with DOX for 16 hours in the presence or absence of MG132 plus or minus the Chk1 inhibitors UCN01 and AZD7762. Cell extracts were prepared, 30 μg resolved by SDS-PAGE, and analysed by western blotting using the indicated antibodies. Upper and lower panels are derived from the same western blots (see Supplementary Information for original images). (**b**) The indicated DT40 cell lines were treated with DOX for 16 hours plus or minus MG132 to induce the expression of Chk1-L393P, and the Chk1-L393P/KR and Chk1-L393P/S345A double mutant proteins (see text for additional explanation) under conditions where the proteasome is active or inhibited. Cell extracts were prepared, 30 μg resolved by SDS-PAGE, and analysed by western blotting using the indicated antibodies. Samples in the upper and lower panels respectively are derived from two western blots run, processed and imaged in parallel in each case (see Supplementary Information for original images). (**c**) Chk1-L393P protein expression was induced by treatment with DOX for 16 hours followed by further treatment with cycloheximide (CHX) for 1, 3, and 6 hours to block protein synthesis in the presence or absence of the Chk1 inhibitor AZD7762. Cell extracts were prepared, 30 μg resolved by SDS-PAGE in triplicate, and analysed by western blotting using antibodies recognising total Chk1 and β-Actin. Shown is the mean and standard deviation of three independent determinations of the amount of Chk1 remaining for each treatment using β-Actin to correct for loading. Control values at t = 0 have been converted to 100% to facilitate comparison of relative protein stability under each condition. (**d**) Chk1-L393P and Chk1-L393P/KR protein expression was induced by treatment with DOX for 16 hours followed by further treatment with cycloheximide (CHX) for 1, 3, and 6 hours to block protein synthesis. Cell extracts were prepared, 30 μg resolved by SDS-PAGE in triplicate, and analysed by western blotting using antibodies recognising total Chk1 and β-Actin. Shown is the mean and standard deviation of three independent determinations of the amount of Chk1 remaining for each treatment using β-Actin to correct for loading. Control values at t = 0 have been converted to 100% to facilitate comparison of relative protein stability.

These experiments established that Chk1 T378/382 phosphorylation occurs *in vivo*, however we considered it necessary to verify that this in fact represented auto-phosphorylation. To this end we first treated cells expressing the constitutively active Chk1-L393P mutant with MG132, alone or in combination with two selective Chk1 inhibitors, UCN-01 and AZD7762. As shown in Fig. 5a, whereas MG132 treatment alone resulted in strong accumulation of T378/382-phosphorylated Chk1-L393P, this signal was almost completely eliminated when cells were also exposed to UCN-01 and AZD7762, although it was notable that the mutant protein was stabilised to a similar degree. Thus, two chemically distinct inhibitors of Chk1 efficiently block phosphorylation of T378/382 *in vivo*. We also used a previously described kinase inactive form of the constitutively active KA1 mutant, Chk1-L393P, Chk1-L393P/KR^14^. Chk1-L393P/KR contains the activating KA1 domain L393P mutation in combination with a substitution of arginine for lysine 38 (K38R) within the kinase domain, which ablates Chk1 kinase catalytic activity^14^. The Chk1-L393P/KR double mutant protein was also stabilised by treatment with MG132 (Fig. 5b), however phosphorylation of T378/382 was not observed. Thus, two independent lines of evidence indicate that active mutants of Chk1 auto-phosphorylate at T378/382 *in vivo*.

To determine whether T378/382 auto-phosphorylation might depend on prior modification of the key ATR regulatory site, S345, we took advantage of a second double mutant, Chk1-L393P/S345A, in which the KA1 mutation is combined with a substitution of a non-phosphorylatable alanine residue for S345^14^. As shown in Fig. 5b, unlike Chk1-L393P/KR, the Chk1-L393P/S345A mutant protein was observed to auto-phosphorylate vigorously when proteasomal protein degradation was inhibited with MG132. Thus, T378/382 phosphorylation does not require prior modification of S345, at least in the context of a constitutively active KA1 domain mutant such as Chk1-L393P.

### T378/382 auto-phosphorylation accelerates proteasomal degradation of Chk1

That the T378/382 auto-phosphorylated forms of the activated mutants Chk1-L393P and Chk1-F455P were selectively stabilised by MG132 suggested that this modification might signal proteasomal degradation. To test this we performed two experiments. In the first, we treated DOX-induced cells expressing Chk1-L393P with cycloheximide in the presence or absence of AZD7762 to block T378/382 auto-phosphoryation. As shown in Fig. 5c, this revealed that the rate of degradation of Chk1-L393P was slowed in the presence of AZD7762. Next, we compared the relative stability of Chk1-L393P with its kinase-dead counterpart, Chk1-L393P/KR, which is unable to auto-phosphorylate *in vivo*. Consistent with the effects of AZD7762, we observed that Chk1-L393P/KR was degraded significantly more slowly than Chk1-L393P (Fig. 5d). We deduce from these results that T378/382 auto-phosphorylation accelerates rapid proteasomal degradation of the constitutively active Chk1-L393P mutant. It was notable however that even in the absence of auto-phosphorylation the Chk1-L393P mutant protein was still relatively unstable (Fig. 5c,d), indicating that auto-phosphorylation is not strictly obligatory for degradation.

### T378/382 auto-phosphorylation of Chk1-WT is not augmented by genotoxic stress

**Figure 6:**
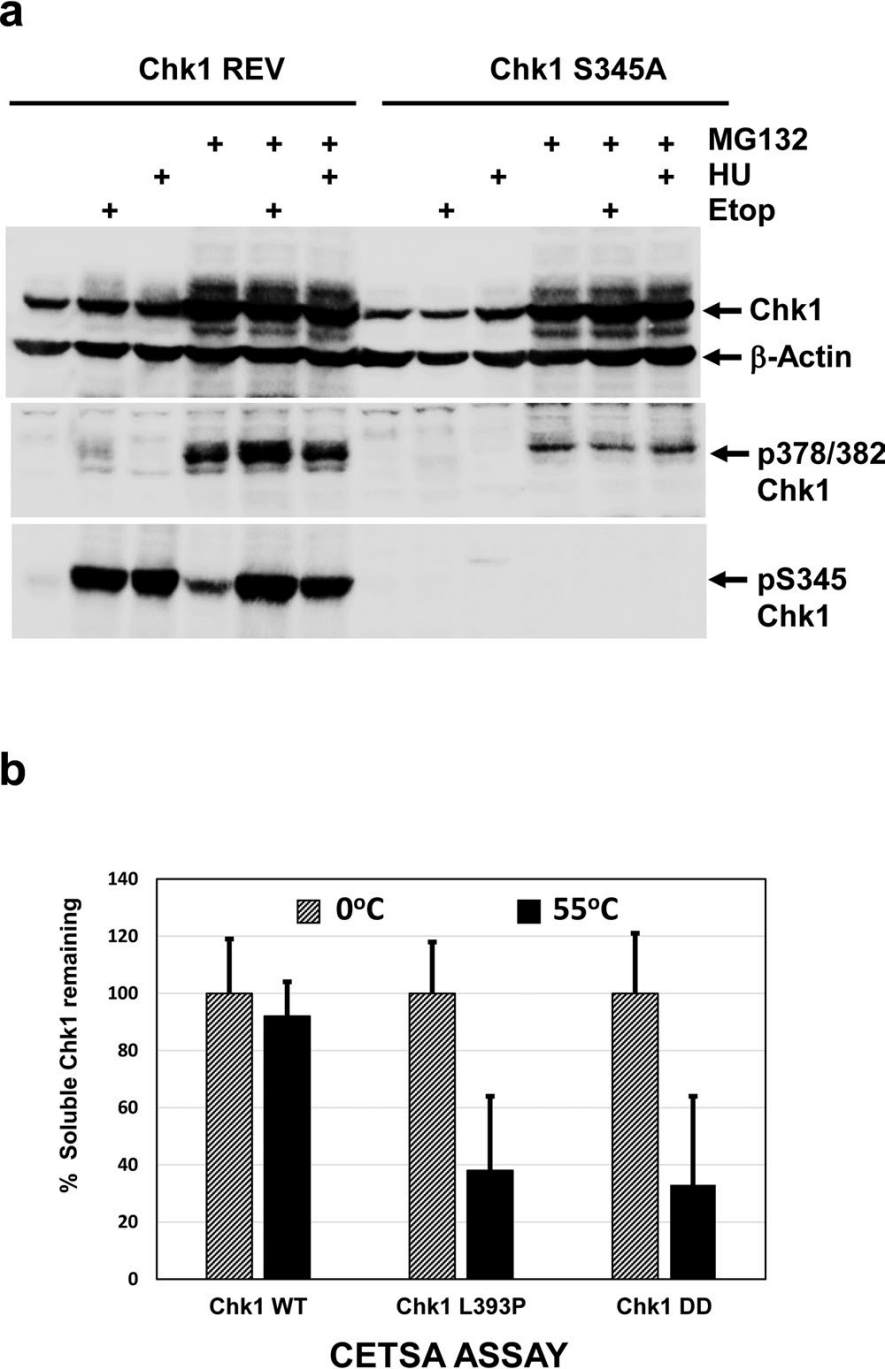
Chk1 T378/382 auto-phosphorylation is not augmented by DNA damage or DNA synthesis inhibition. (**a**) Chk1 REV DT40 cells stably expressing Chk1-WT, or Chk1-S345A DT40 cells stably expressing a mutant where alanine replaces S345 (see text for additional explanation), were treated for 16 hours with ETOP and HU in the presence or absence of MG132. Cell extracts were prepared, 30 μg resolved by SDS-PAGE, and analysed by western blotting using the indicated antibodies. Samples were resolved on three separate western blots (see Supplementary Information for original images). (**b**) CETSA analysis of the thermal stability of the Chk1-WT, Chk1 L393P, and Chk1-DD proteins in cell extracts. Protein expression was induced by treatment with DOX for 16 hours and native, soluble whole cell extracts prepared^14^. Portions of each extract were heated at 55 °C for 5 minutes or retained on ice. Insoluble proteins were then removed by centrifugation and 30 μg resolved by SDS-PAGE in triplicate and analysed by western blotting using antibody recognising total Chk1. Shown is the mean and standard deviation of the percentage of soluble Chk1 remaining after treatment at 55 °C.

To determine whether T378/382 auto-phosphorylation was affected by DNA damage or DNA synthesis inhibition, we cultured Chk1 knockout cells stably expressing Chk1-WT^6^ overnight in the presence or absence of MG132 together with the genotoxic agents etoposide (ETOP) and Hydroxyurea (HU). As shown in Fig. 6a, overnight treatment with ETOP and HU alone did not result in significant levels of T378/382-phosphorylated Chk1-WT compared to that obtained with MG132 alone, although as expected, both induced strong phosphorylation of Chk1 at S345. Reasoning that this might be because Chk1-WT was rapidly degraded subsequent to auto-phosphorylation we treated cells overnight with ETOP or HU together with MG132 to inhibit the proteasome. Surprisingly, co-treatment with ETOP or HU and MG132 did not increase the level of T378/382 auto-phosphorylation over treatment with MG132 alone, despite high levels of S345 phosphorylation indicative of Chk1 activation by ATR. To further investigate the potential relationship between T378/382 auto-phosphorylation and S345 phosphorylation, we repeated this analysis using Chk1 knockout cells stably expressing Chk1-S345A, a mutant in which S345 is replaced by alanine and which cannot be phosphorylated by ATR at this site^6^. As shown in Fig. 6a, T378/382 auto-phosphorylation of Chk1-S345A was readily detected after treatment with MG132, although the level was significantly lower than observed with Chk1-WT. As with Chk1-WT, Chk1-S345A auto-phosphorylation at T378/382 was not augmented by treatment with ETOP or HU in combination with MG132, whilst as previously described^6^, no signal was detected using antibody specific for Chk1 phosphorylated at S345 (Fig. 6a). We conclude from these data that ATR-mediated Chk1 S345 phosphorylation is not obligatory for T378/382 auto-phosphorylation and that this latter modification, surprisingly, it is not stimulated by genotoxic stress.

Finally, we investigated whether the Chk1-L393P and phospho-mimetic Chk1-DD mutations might result in conformational changes that could contribute to activation and degradation using the “Cellular Thermal Shift Assay” (CETSA)^17^. The CETSA technique has been used to measure the interaction of proteins with drugs or ligands by determining the relative thermal stability of the bound and unbound forms in cell extracts^17^, however we reasoned that it should also detect conformational changes in stability resulting from structural mutations. To test this, we determined the maximum temperature (55 °C) at which Chk1-WT remained soluble in cell extracts (see Materials and Methods for details), and then compared the relative thermal stability of the active Chk1-L393P and Chk1-DD mutants at this temperature. As shown in Fig. 6b, both the KA1 domain structural mutant Chk1-L393P and the phospho-mimetic Chk1-DD mutant showed significantly elevated levels of heat-induced precipitation at 55 °C, indicative of reduced thermal stability that we attribute to mutationally-altered structural conformation compared to Chk1-WT^14^.

## Discussion

Here, we identify two novel Chk1 auto-phosphorylation sites, T378/382, and show that modification of these sites is linked to Chk1 activation and proteasomal degradation. T378/382 exist within optimal consensus sequences for Chk1 phosphorylation unlike the previously identified auto-phosphorylation site at S296^9^. It is conceivable therefore that T378/382 auto-phosphorylation might occur with distinct kinetics or under different conditions to that occurring at S296, although we have not addressed this issue directly. Importantly, the region of Chk1 containing T378/382 has previously been linked to Chk1 ubiquitination and proteasomal degradation^12^. Ubiquitination of activated, S345-phosphorylated Chk1 by the CRL1–SKP1–Fbx6 complex was shown to be mediated by a degron-like region contained between amino acids 368–421 within the KA1 domain^12^. Although the exact amino acid sequence of the degron was not established, it is striking that mutation of three lysine residues (K372/376/379) that lie adjacent to or overlapping T378/382 within the conserved motif (Fig. 1a) severely impaired both Fbx6 binding and ubiquitination of Chk1^12^. It therefore seems highly likely that Chk1 T378/382 auto-phosphorylation in fact occurs within this previously defined degron region^12^.

This previous study also proposed that inactive Chk1 was stable because an intramolecular interaction between the C-terminal regulatory and kinase domains both suppressed kinase activity and rendered the degron inaccessible to the CRL1–SKP1–Fbx6 complex^12^. This interaction is now known to be mediated specifically by the KA1 domain^7^, and it seems likely that mutations designed to disrupt this structure, such as Chk1-L393P^14^, result in a conformational change that both de-represses catalytic activity and exposes the degron. That the Chk1-L393P mutant exhibits reduced thermal stability compared to Chk1-WT in CETSA assays and auto-phosphorylates vigorously both *in vitro* and *in vivo* strongly supports this interpretation. Thus, we believe that conformational exposure of the degron likely explains why Chk1-L393P is inherently less stable than Chk1-WT. Why T378/382 auto-phosphorylation within the degron region further accelerates proteasomal degradation is less clear. Two possible explanations can be considered; phosphorylation of T378/382 might increase the inherent affinity of the degron for the CRL1–SKP1–Fbx6 or potentially other ubiquitin ligase complexes, or alternatively, might induce further conformational changes that maximise degron accessibility. Characterisation of the Chk1 degron will be necessary to distinguish between these possibilities.

The Chk1-DD mutant protein also shows lower thermal stability than Chk1-WT in cell extracts, indicating that these phospho-mimetic mutations result in a conformational change. Inspection of the recently described crystal structure of the Chk1 KA1 domain^7^ reveals that T378/382 are located within a hydrophobic pocket thought to play an important role in stabilising the overall KA1 domain fold. We hypothesise therefore that phosphorylation of T378/382, or their replacement with charged phospho-mimetic residues, is sufficient to disrupt KA1 domain-mediated repression of the kinase domain and render the degron accessible. This, coupled with enhanced degron potency due to the presence of charged phospho-mimetic aspartic acid residues in place of unphosphorylated T378/382, provides a logical explanation for why the Chk1-DD mutant is degraded more rapidly than Chk1-L393P, even though both appear to be equivalent in terms of their constitutive biological activity (Fig. 2)^14^.

That Chk1-DD is constitutively active strongly suggests that auto-phosphorylation of Chk1 T378/382 is somehow linked to kinase activation. Consistent with this, small amounts of Chk1-WT clearly undergo auto-phosphorylation, we assume reflecting activation, during unperturbed cell growth. Perplexingly however, and in marked contrast to what has been reported for S296^9^, DNA damage or DNA synthesis inhibition did not stimulate auto-phosphorylation of T378/382 in Chk1-WT, even when the proteasome was inhibited and S345 phosphorylation was efficiently induced. We infer from this that T378/382 auto-phosphorylation is not part of the canonical mechanism of Chk1 activation in response to genotoxic stress. This raises the question of what controls Chk1 T378/382 auto-phosphorylation and what the function of this form of Chk1 might be? Although we cannot answer these questions definitively at this time, the location of these sites within, and the potential for their modification to disrupt, the Chk1 KA1 domain is intriguing. Although their properties and functions are diverse, several KA1 domains are known to bind and inhibit their cognate kinase domains, including that of Chk1. In some cases KA1-binding trans-regulatory molecules, such as the SOS3 protein for the plant kinase SOS2^18^ or phospholipids in the case of MARCK1^19^, can relieve or de-repress auto-inhibition by dissociating the intra-molecular interaction between the kinase and KA1 domains. Thus, amino acids in the MARK1 KA1 domain that mediate interaction with the kinase domain are also those that bind phospholipids in a mutually exclusive fashion^19^. Interestingly, this mechanism can provide a means of coordinating kinase activation with targeting to a specific subcellular localisation such as membranes^20^. We speculate therefore that Chk1 T378/382 auto-phosphorylation may occur when auto-inhibition imposed by the KA1 domain is relieved by interaction with an as yet unidentified trans-regulator, and that the modification may serve to maintain Chk1 in an active configuration whilst simultaneously promoting proteasomal degradation. Further work will be required to assess this possibility.

## Materials and Methods

### Cell culture and treatments

3T DT40 cells and derived cell lines were grown in Dulbecco’s Modified Eagle’s Medium (DMEM; Invitrogen, Carlsbad, CA, USA) containing 10% tetracycline-free fetal bovine serum and 1% chicken serum as described previously^14^. 3T cells are a derivative of the Chk1 knockout DT40 cell line described previously that contain a single, stably integrated copy of the pFRT/lacZeo acceptor plasmid of the Invitrogen “Flp-In” system together with the pcDNA6/TR plasmid encoding the Tet repressor^14^. The Chk1 mutant bearing phospho-mimetic aspartic acid residues in place of T378 and T 382 (Chk1-DD) was cloned into the pcDNA5/FRT donor plasmid and co-transfected with the Flp recombinase-encoding plasmid pOG44 leading to stable integration of the mutants into the pFRT/lacZeo expression cassette under the control of a Tet-regulated promoter. Chk1 protein expression was induced by treating cultures with 50 ng/ml doxycycline (DOX) for the required time (usually 16 hours). To inhibit the proteasome cells were treated with 5 μM MG132 (Calbiochem, San Diego, USA) and to induce DNA damage or DNA synthesis inhibition cells were treated with 20 ng/ml etoposide Cayman Chemical (Hamburg, Germany) or 2 mM HU (Alfa Aesar, Karlsruhe, Germany) for 16 hours respectively. To inhibit Chk1 cells were treated with AZD7762 (Cayman Chemical) or UCN01 (Sigma Aldrich, Madrid, Spain) at a final concentration of 1 μM.

### Generation of the anti-phospho-T378/382 Chk1 antibody

The anti-p378/382 serum was generated and purified by Eurogentec (Seraing, Belgium). Briefly, rabbits were immunised with a 12 amino acid di-phosphorylated peptide spanning Chk1 T378/382 (VKRMpTRFFpTKLD). The resulting immune serum was purified by affinity chromatography on a column containing the di-phosphopeptide, eluted, and subsequently depleted of non-phospho-specific antibodies by affinity chromatography on a column bearing the corresponding non-phosphorylated peptide. Subsequent tests (Fig. 1b) demonstrated that the resulting purified serum was highly specific for Chk1 phosphorylated at T378/382 with no detectable affinity for non-phosphorylated Chk1 and did not recognise the Chk1-DD mutant that lacks these threonine residues (Supplementary Fig. 1). Anti-pT378/382 was also able to specifically immune-precipitate the auto-phosphorylated but not the non-modified form of Chk1 from cell extracts (Supplementary Fig. 2).

### Site-directed mutagenesis

The Chk1-DD mutant was generated using a “Quickchange” site-directed mutagenesis kit (Stratagene, La Jolla, CA, USA). The sequence of the mutagenic primers used to make the amino acid substitutions are available on request. Mutations were verified by sequencing prior to stable transfection into 3T cells and functional analysis.

### Flow cytometry

Cells were fixed in 70% ethanol overnight at 4 °C. Fixed cells were incubated in phosphate-buffered saline containing 0.1% Tween 20 (PBT) with polyclonal anti-phospho-serine 10 histone H3 antibodies to identify mitotic cells (pH3; Santa Cruz Biotechnology, Santa Cruz, CA, USA; sc-8656; also used for western blotting) followed by Alexa 488-conjugated secondary antibody (Life Technologies, Madrid, Spain) for 1 hour as described previously^14^. Cells were then counterstained in PBS containing 50 μg/ml propidium iodide and 250 μg/ml RNaseA for a further hour. Samples were analysed using a MACSQuant Analyzer and software (Miltenyi Biotech, Germany).

### Western blotting and kinase assays

Cells were washed once in ice-cold phosphate-buffered saline (PBS), lysed in lysis buffer (LB)^14^, resolved by SDS-PAGE gel electrophoresis, and analysed by western blotting using antibodies recognising total Chk1 and Chk1 phosphorylated on T378/382 or S345 as described previously^14^. Antibody against PCNA (PC10) was from Santa Cruz Biotechnology and against B-actin from Genscript (Piscataway, NJ, USA). Western blot images were captured using a ImageQuant LAS 4000 mini and quantified using ImageQuant TL software (GE Healthcare Life Sciences). Kinase reactions using recombinant GST-Chk1 purified from insect cells (Merck, Darmstadt, Germany) were carried out plus or minus 100 μM ATP at 30 °C for 30 mins as previously described^14^.

### CETSA assays

Cell extracts containing native, soluble Chk1 proteins were prepared as described previously using Whole Cell Extract buffer (WCE)^14^ and ranging experiments performed where extracts containing Chk1-WT were heated for 5 mins at increasing temperatures (2 °C increments) from 40–60 °C using a Grant QBD2 heating block^17^. After heating, insoluble material was removed by centrifugation and the percentage of Chk1-WT that remained soluble at each temperature determined by western blotting. Chk1-WT was found to be almost completely soluble after heating at 55 °C but became increasingly insoluble at higher temperatures. 55 °C was therefore selected as the temperature at which to compare the thermal stability of Chk1-WT with the mutant derivatives Chk1-L393P and Chk1-DD.

## Electronic supplementary material

Supplementary Information
