# Supplementary material for: Chk1 KA1 domain auto-phosphorylation stimulates biological activity and is linked to rapid proteasomal degradation
Source: Sci Rep. 2018 Dec 3;8:17536. doi: 10.1038/s41598-018-35616-9 (PMC6277497; doi:10.1038/s41598-018-35616-9)
Supplement: Supplementary file 1 — Supplementary Information [file 41598_2018_35616_MOESM1_ESM.pdf]

**Supplementary Information**

# Chk1 KA1 domain auto-phosphorylation stimulates biological activity and is linked to rapid proteasomal degradation


Eun-Yeung Gong[1#], Beatriz Hernández[1], Jessica Hernández Nielsen[1], Veronique AJ Smits[1], Raimundo Freire[1], and David A Gillespie[2*]

[1]Unidad de Investigación, Hospital Universitario de Canarias, Ofra s/n, La Cuesta, La Laguna 38320, Tenerife, Spain

[2]Instituto de Tecnologías Biomédicas, Centro de Investigaciones Biomédicas de Canarias, Facultad de Medicina, Campus Ciencias de la Salud, Universidad de La Laguna, La Laguna 38071, Tenerife, Spain

*Corresponding author. Email: dgillesp@ull.edu.es Tel: +34 922 678 111 Fax: +34 922 647 112

# Nano-Bio Resources Center, Sookmyung Women's University, Chungpa-Dong 2-Ka, Yongsan-ku, Seoul 04310, Republic of Korea


Running title: Chk1 regulation by auto-phosphorylation


Key words: Chk1, auto-phosphorylation, DNA damage, cell cycle arrest, proteasomal protein degradation.

# Supplementary Figure 1

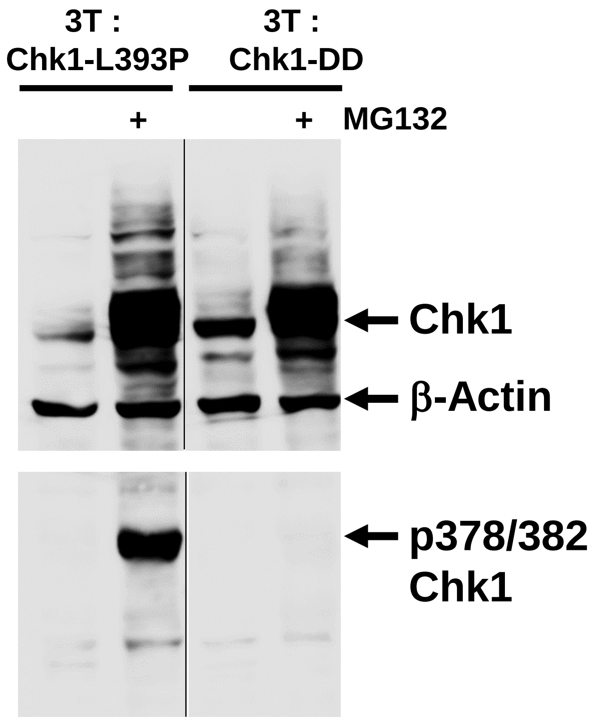


**Recognition of auto-phosphorylated Chk1 by anti-pT378/382 antiserum is dependent on threonines 378 and 382**

3T: Chk1-L393P and 3T: Chk1-DD DT40 cell lines were treated for 16 hours with DOX plus or minus MG132 to inhibit protein degradation via the proteasome. Cell extracts were prepared, 30mg resolved by SDS-PAGE, and analysed by western blotting using antibodies against total Chk1 and b-Actin (upper panel) and antipT378/382 phospho-specific Chk1 antiserum (lower panel). Auto-phosphorylated Chk1-L393 protein is stabilized by MG132 and detected by anti-pT378/382. Chk1-DD is also stabilized by MG132 but is not detected by anti-p378/382. Samples were resolved on two separate western blots (see Supplementary Information for original images).

# Supplementary Figure 2

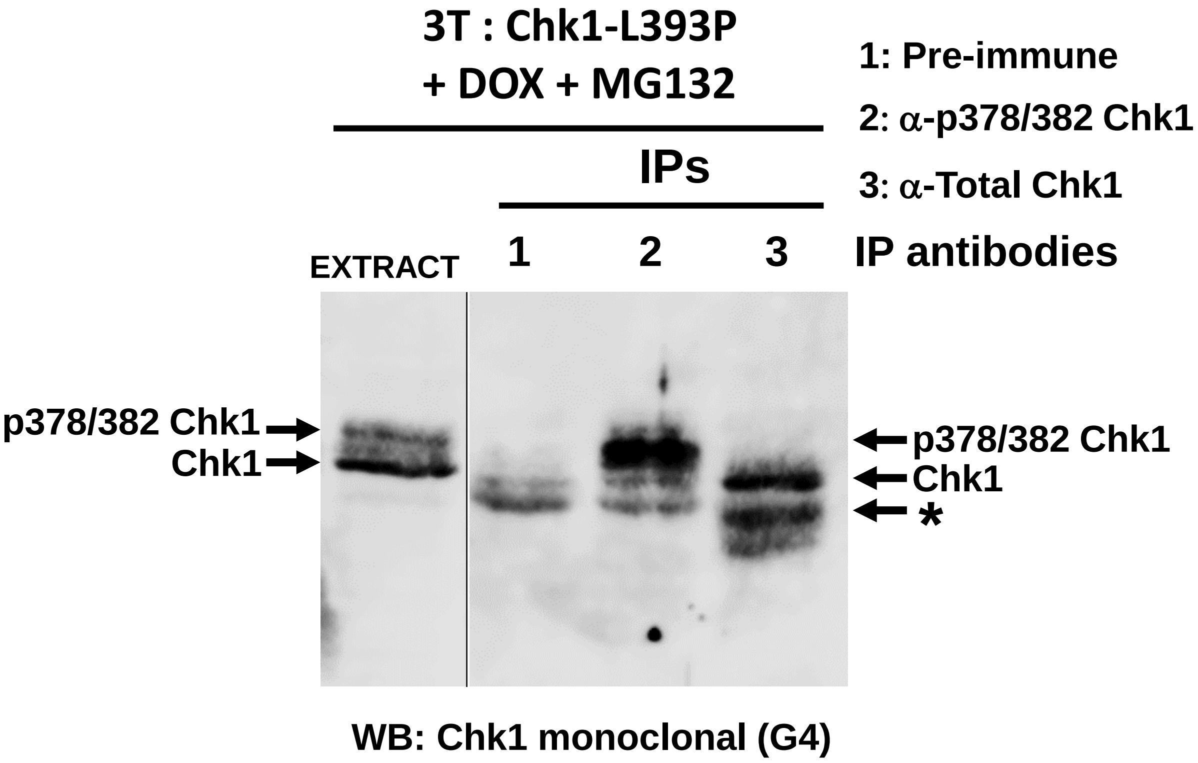


**Anti-pT378/382 antiserum selectively immuno-precipitates auto-phosphorylated Chk1**

3T: Chk1-L393P DT40 cell lines were treated for 16 hours with DOX plus MG132 to inhibit protein degradation via the proteasome. Whole cell extracts were prepared, and equal portions immuno-precipitated with pre-immune serum (PI), anti-pT378/382, and a polyclonal antiserum against total Chk1. Immuno-precipitates were resolved by SDS-PAGE, transferred to nitrocellulose, and the membrane probed with a monoclonal antibody against Chk1 (G4) in combination with protein A-HRC conjugate for detection (see Supplementary Information for original images). The shifted, auto-phosphorylated form of Chk1 that is stabilized by MG132 is selectively immuno-precipitated by antip378/382 antiserum. Asterisk indicates precipitating antibody heavy chains that are detected weakly by the protein HRP conjugate.

# Figure 1: Uncropped Images

Cropped areas in Figures boxed in dashed lines.

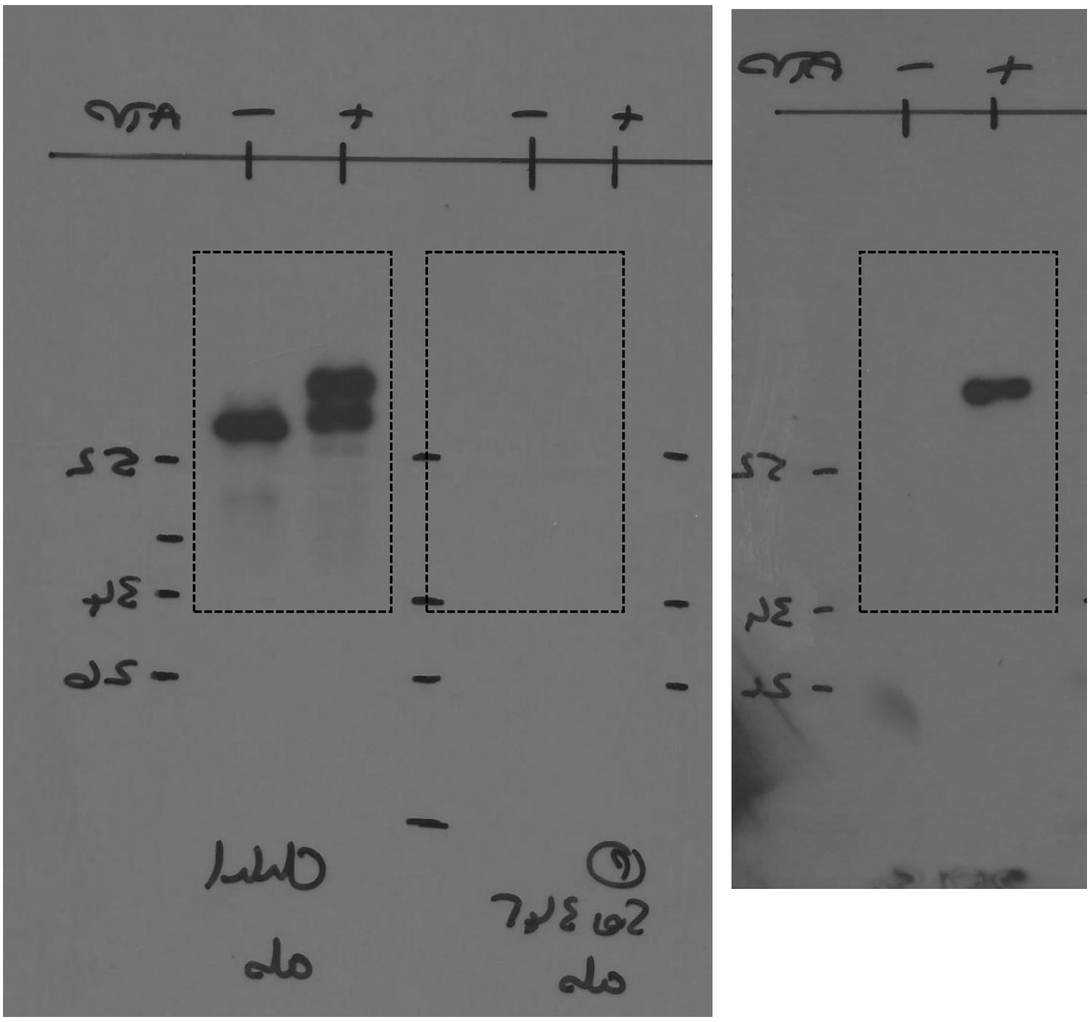

3 replicate western blot panels

# Figure 2: Uncropped Images

GEL 1

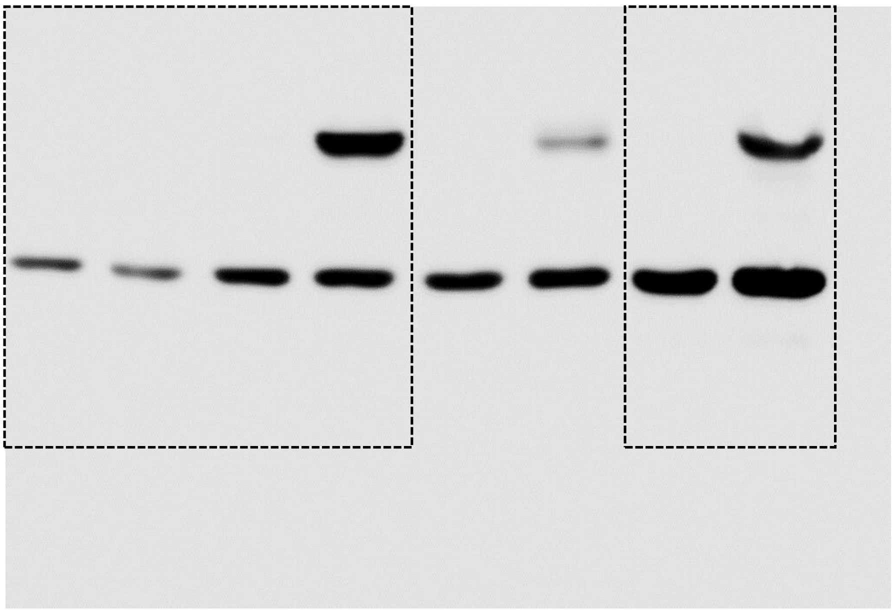

GEL 2

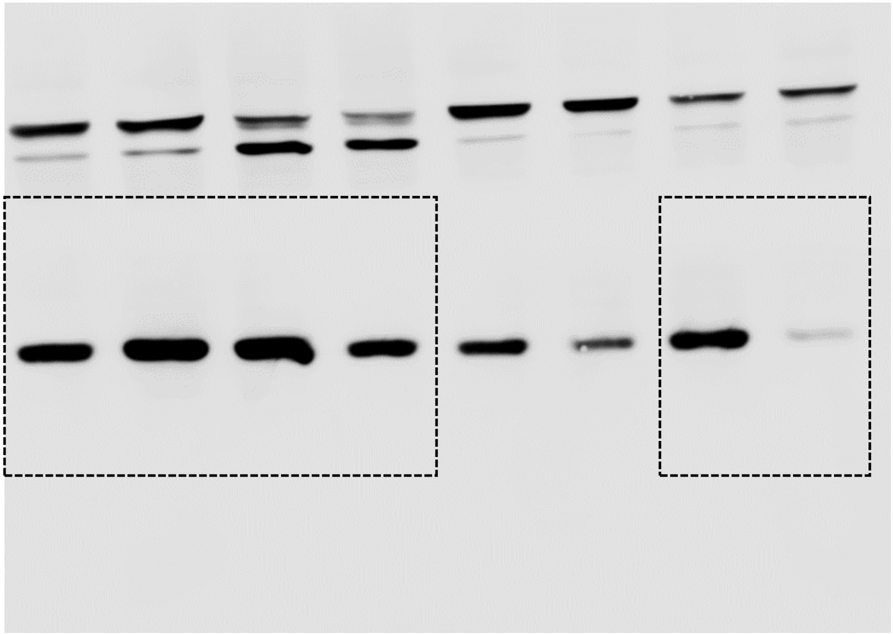

# Figure 3: Uncropped Images

GEL 1

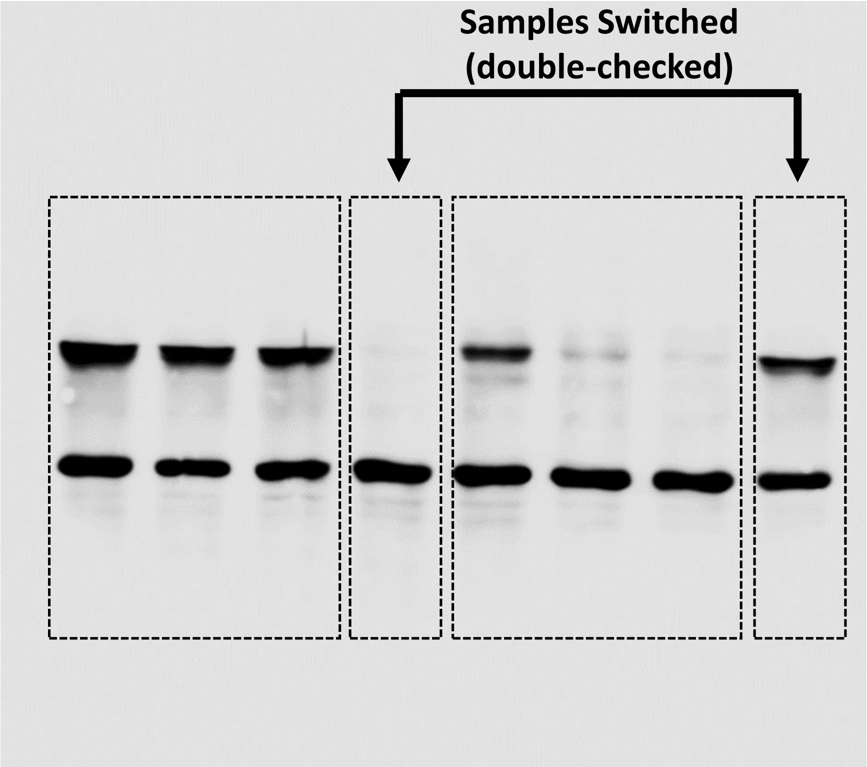


GEL 2

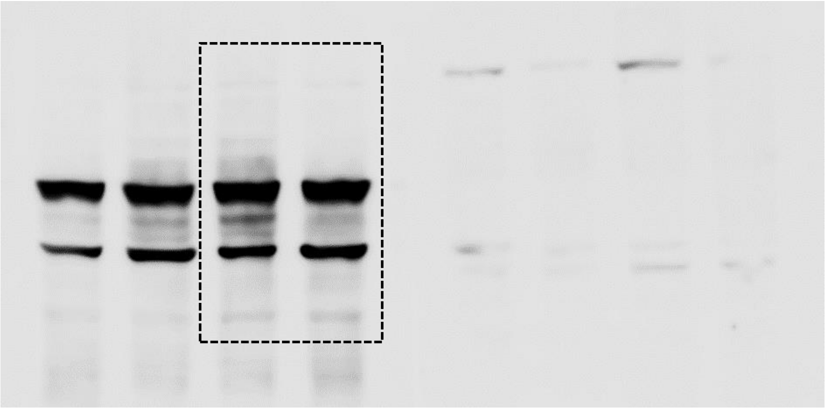

GEL 3

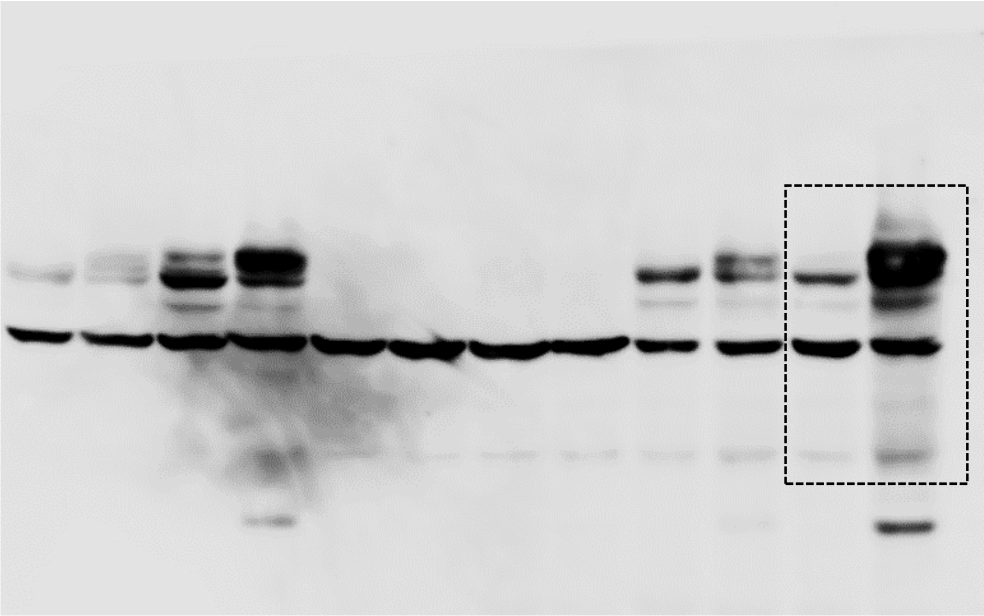

# Figure 4: Uncropped Images

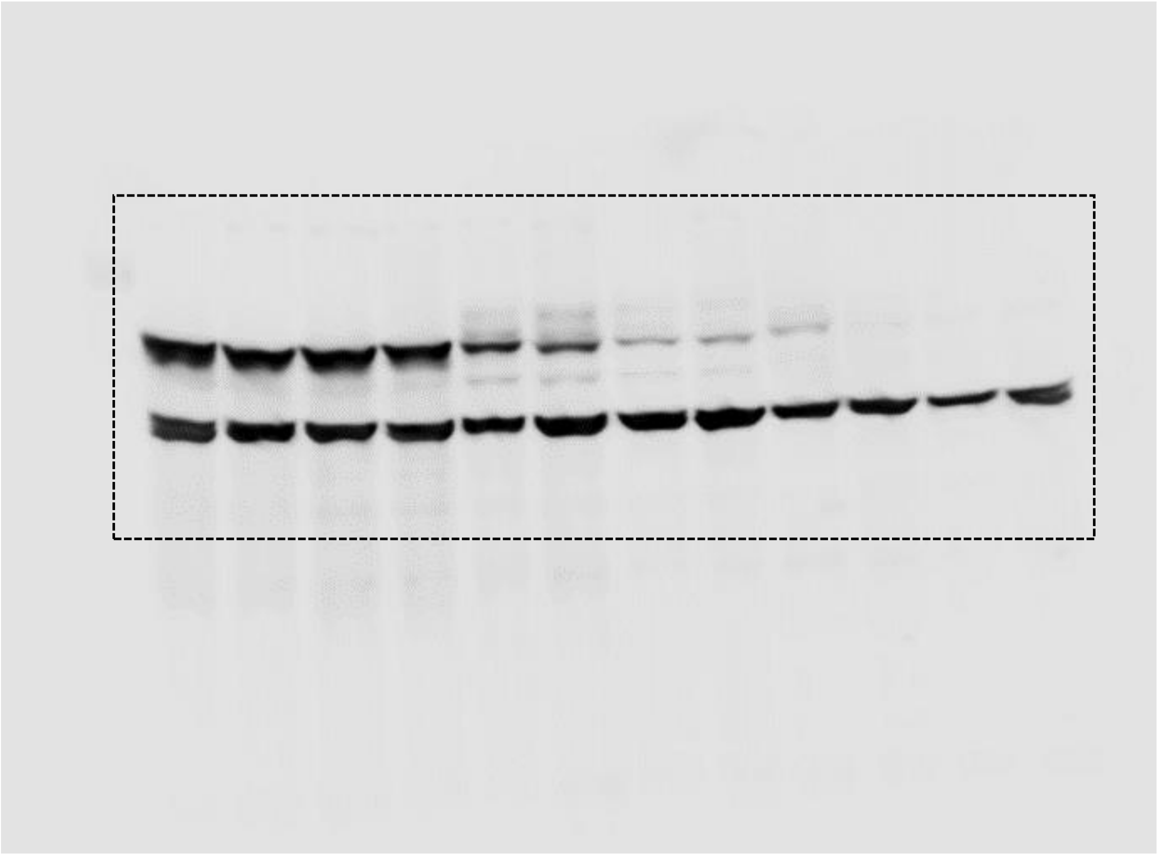

GEL 1

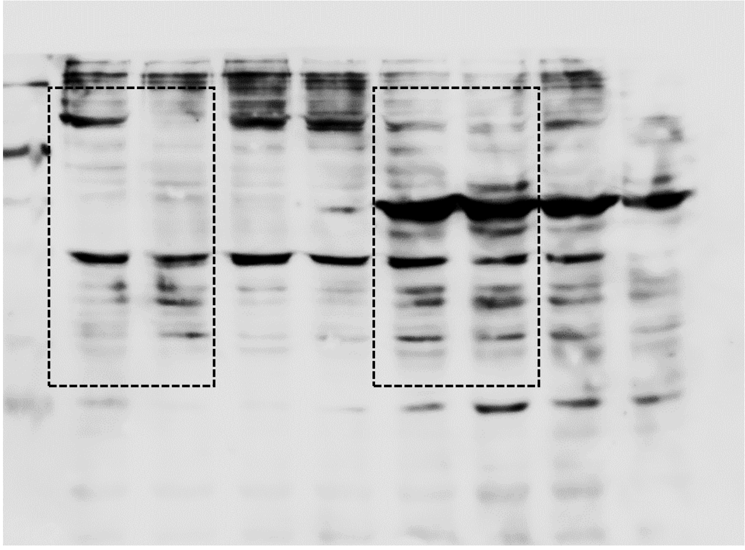

GEL 2

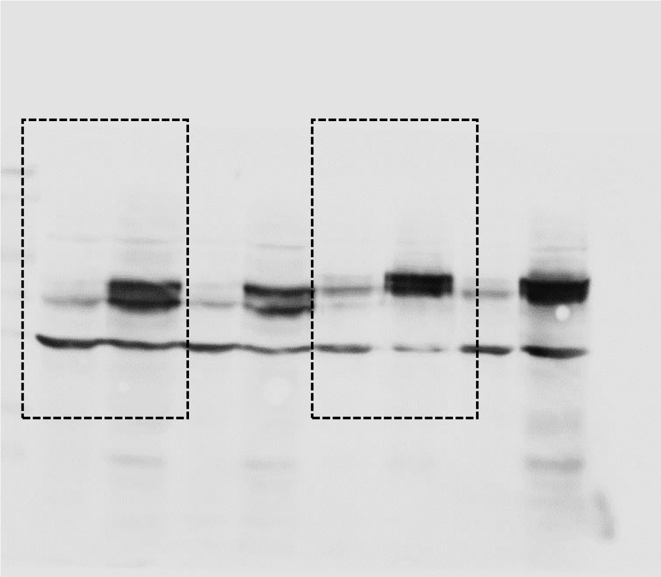

GEL 3

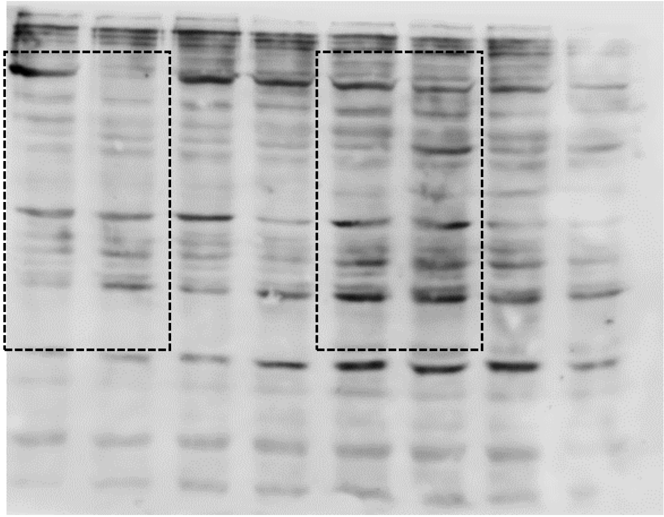

GEL 4

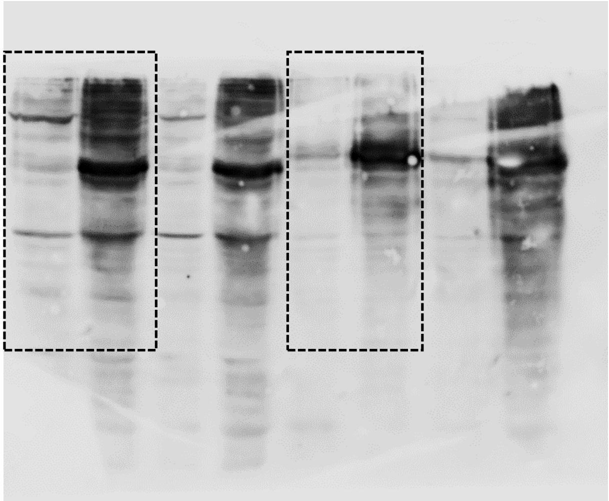

GEL 5

# Figure 5A: Uncropped Images

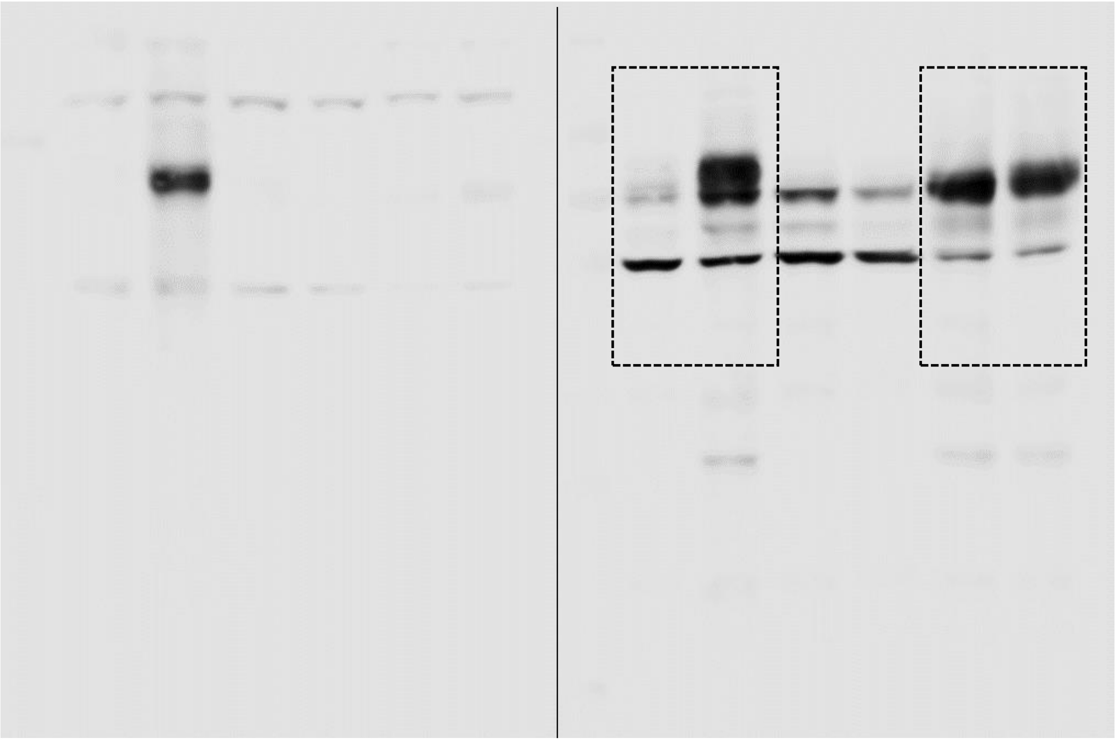

GEL 1 Short exposure GEL 2

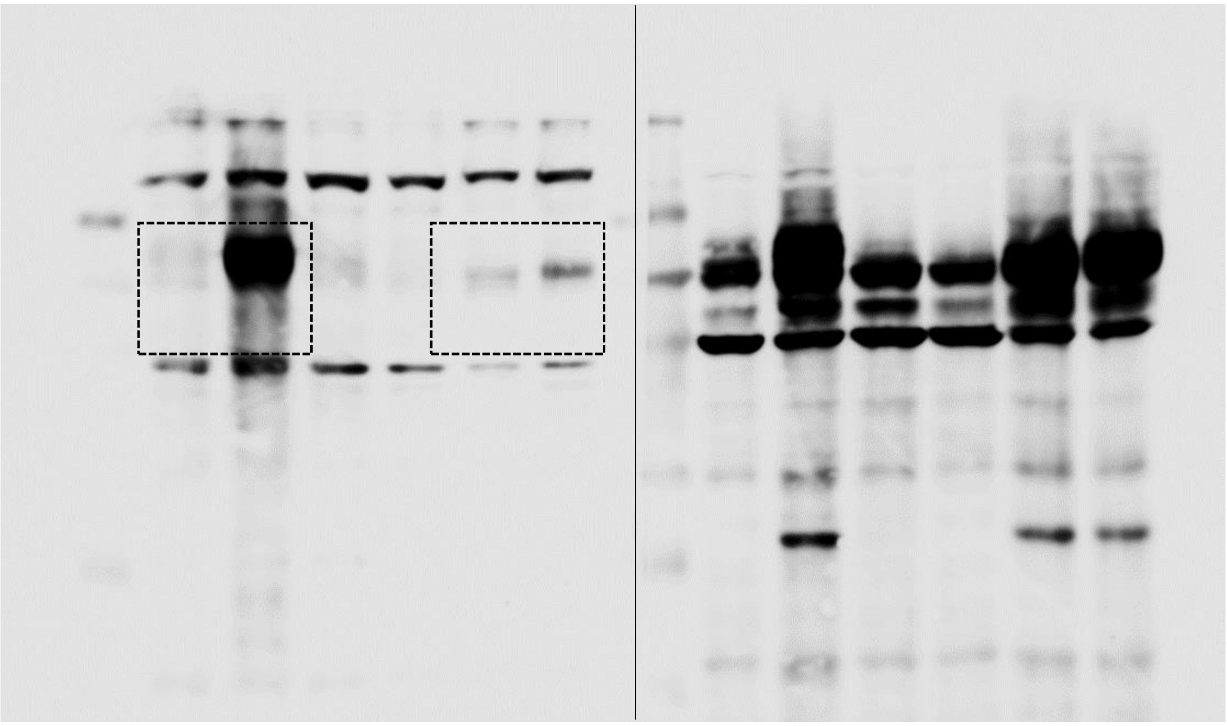

GEL 1 Long exposure GEL 2

# Figure 5B: Uncropped Images

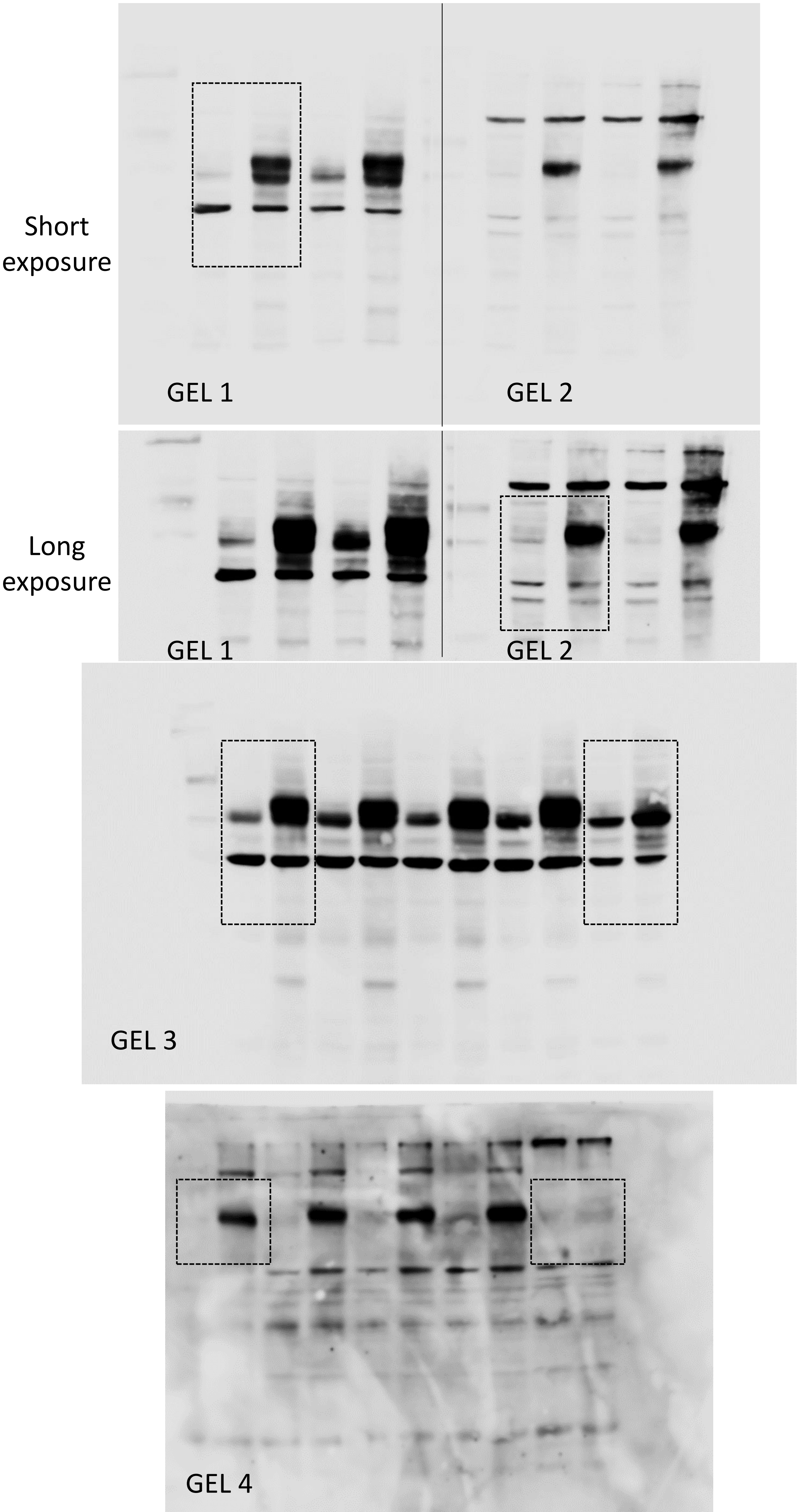

# Figure 6: Uncropped Images

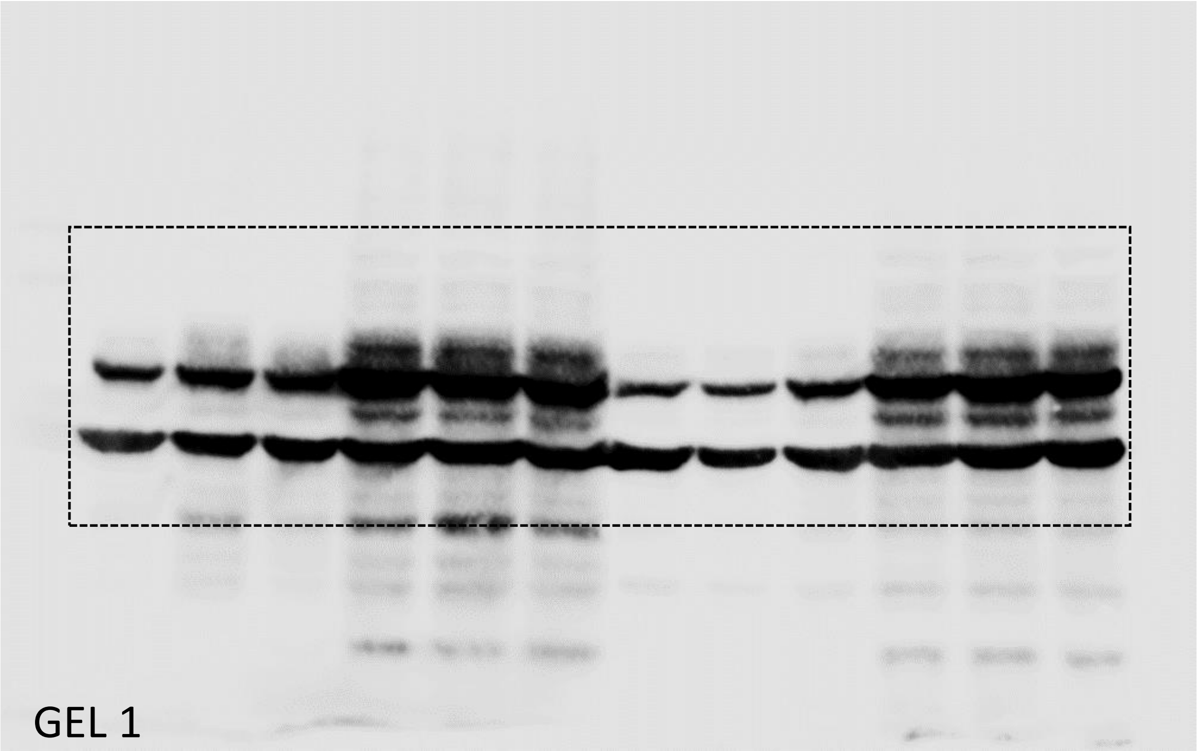


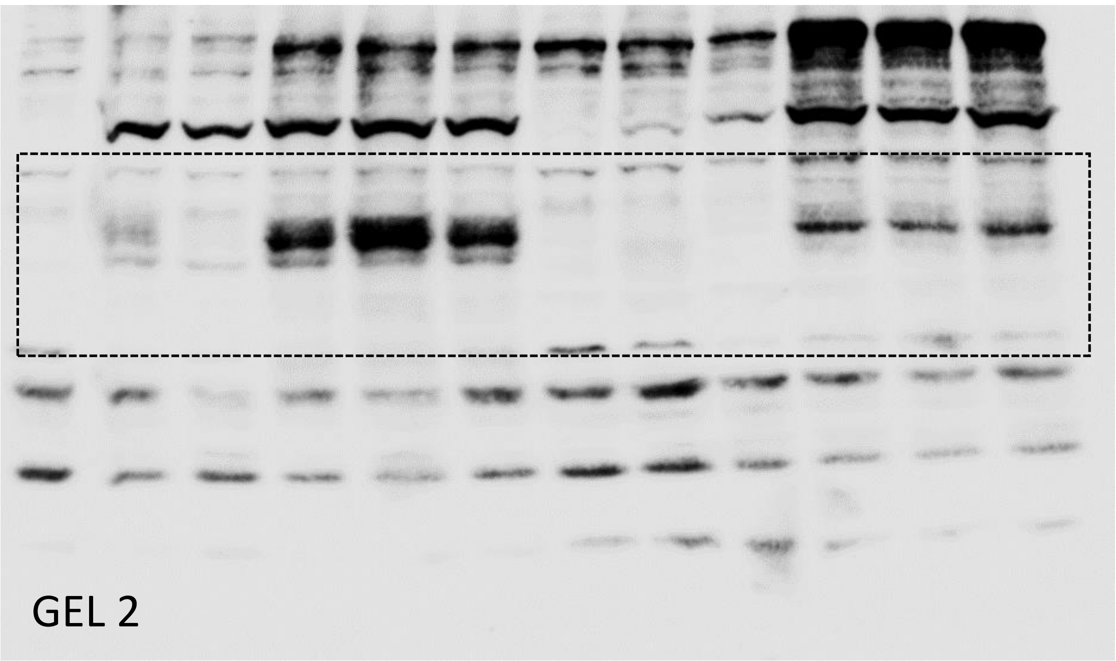


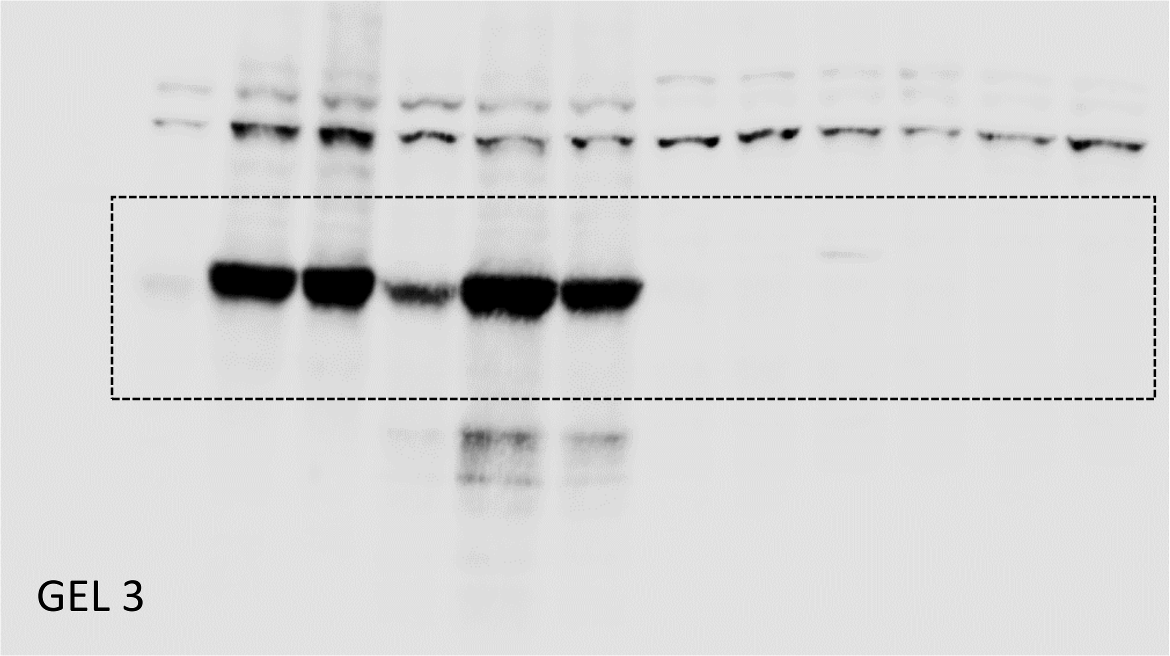

# Supplementary Fig. 1: Uncropped Images

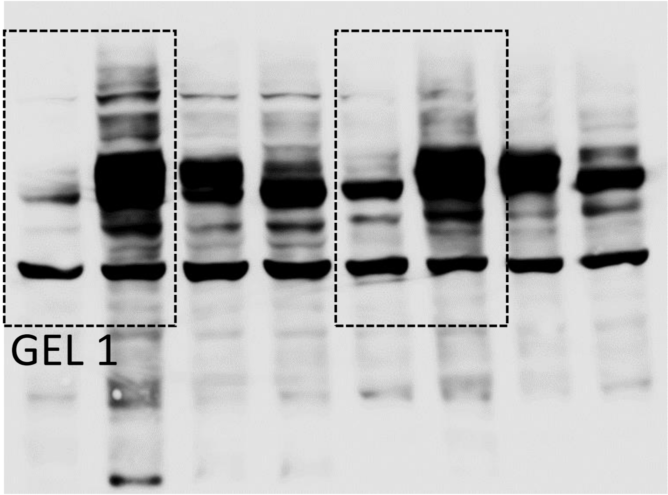


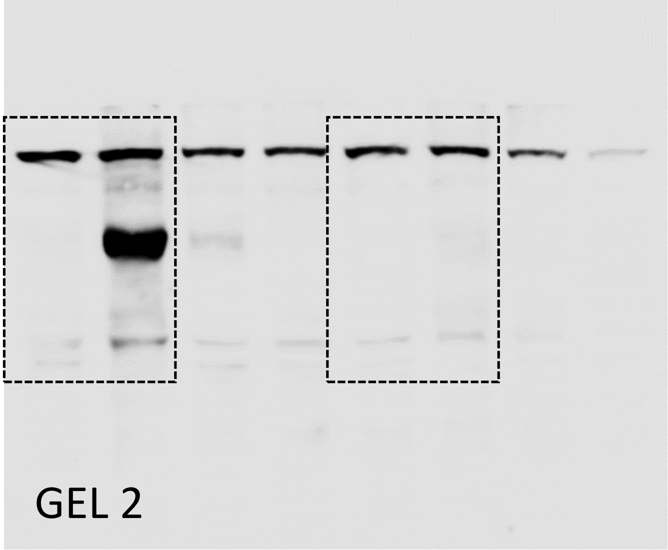


# Supplementary Fig. 2: Uncropped Images

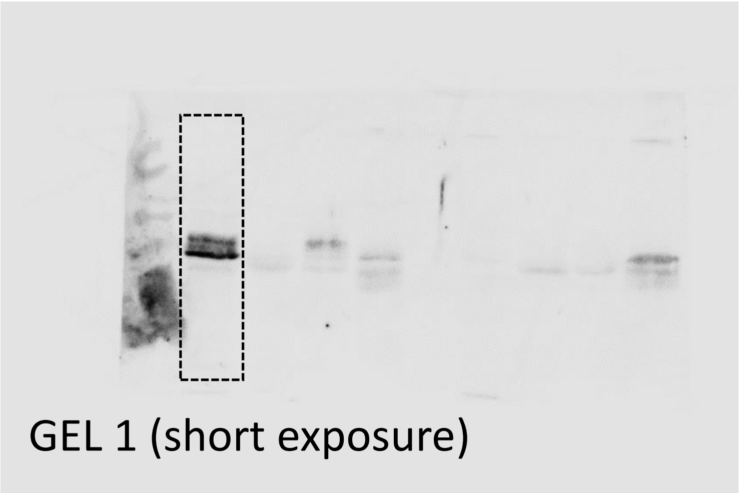


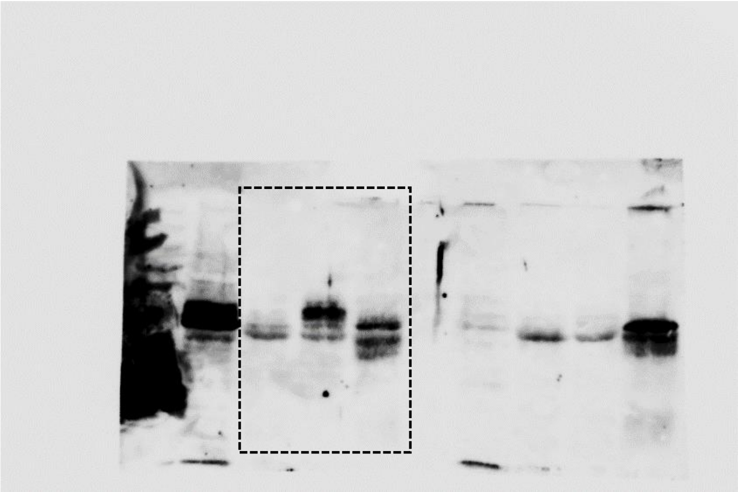

GEL 1 (long exposure)
